# Combining amplicon sequencing and metabolomics in cirrhotic patients highlights distinctive microbiota features involved in bacterial translocation, systemic inflammation and hepatic encephalopathy

**DOI:** 10.1038/s41598-018-26509-y

**Published:** 2018-05-29

**Authors:** Valerio Iebba, Francesca Guerrieri, Vincenza Di Gregorio, Massimo Levrero, Antonella Gagliardi, Floriana Santangelo, Anatoly P. Sobolev, Simone Circi, Valerio Giannelli, Luisa Mannina, Serena Schippa, Manuela Merli

**Affiliations:** 1 Istituto Pasteur Cenci Bolognetti Foundation, Public Health and Infectious Diseases Department, Sapienza University of Rome, Piazzale Aldo Moro 5, 00185 Rome, Italy; 2 Center for Life NanoScience@Sapienza, Istituto Italiano di Tecnologia, Rome, Italy; 3 Gastroenterology, Department of Clinical Medicine, Sapienza University of Rome, Viale dell’Università 37, 00185 Rome, Italy; 4 INSERM, U1052, Cancer Research Center of Lyon (CRCL), Université de Lyon (UCBL1), Centre Léon Bérard, Lyon, France; 5 Public Health and Infectious Diseases Department, Sapienza University of Rome, Piazzale Aldo Moro 5, 00185 Rome, Italy; 6 Department of Drug Chemistry and Technologies, Sapienza University of Rome, Piazzale Aldo Moro 5, I-00185 Rome, Italy; 7 Magnetic Resonance Laboratory “Annalaura Segre”, Institute of Chemical Methodologies, CNR, via Salaria km 29.300, 00015 Monterotondo, (RM) Italy

## Abstract

In liver cirrhosis (LC), impaired intestinal functions lead to dysbiosis and possible bacterial translocation (BT). Bacteria or their byproducts within the bloodstream can thus play a role in systemic inflammation and hepatic encephalopathy (HE). We combined 16S sequencing, NMR metabolomics and network analysis to describe the interrelationships of members of the microbiota in LC biopsies, faeces, peripheral/portal blood and faecal metabolites with clinical parameters. LC faeces and biopsies showed marked dysbiosis with a heightened proportion of Enterobacteriaceae. Our approach showed impaired faecal bacterial metabolism of short-chain fatty acids (SCFAs) and carbon/methane sources in LC, along with an enhanced stress-related response. Sixteen species, mainly belonging to the Proteobacteria phylum, were shared between LC peripheral and portal blood and were functionally linked to iron metabolism. Faecal Enterobacteriaceae and trimethylamine were positively correlated with blood proinflammatory cytokines, while Ruminococcaceae and SCFAs played a protective role. Within the peripheral blood and faeces, certain species (*Stenotrophomonas pavanii*, *Methylobacterium extorquens*) and metabolites (methanol, threonine) were positively related to HE. Cirrhotic patients thus harbour a ‘functional dysbiosis’ in the faeces and peripheral/portal blood, with specific keystone species and metabolites related to clinical markers of systemic inflammation and HE.

## Introduction

Compositional shifts in the gut microbiota are linked with liver disease^1,2^. In liver cirrhosis (LC), the alteration in gut microbiota is characterized by an overgrowth of potentially pathogenic bacteria and a decrease in beneficial commensal species^3^. The portal system accounts for 75% of the blood reaching the hepatic parenchyma and connects the gut directly to the liver. Bacteria, bacterial fragments and byproducts reach the liver through the portal system and contribute to a condition of chronic inflammation that is involved in the development of many complications in cirrhotic patients^4,5^. The close relationship between complications in patients with liver cirrhosis and a dysbiotic gut microbiota have raised much attention in the last few years. In fact, the physiopathological mechanism involved in complications such as hepatic encephalopathy (HE) and infections^4^, including spontaneous bacterial peritonitis (SBP), is strictly linked to the translocation of enteric bacteria or their products into the systemic circulation, called bacterial translocation (BT)^6,7^. Decreased gut motility, small intestinal bacterial overgrowth (SIBO), impaired intestinal permeability and deficiencies in the local host immune defences are the major mechanisms implicated in the promotion of pathological BT in cirrhosis^8,9^. It was first proposed by Scheline that the gut microbiota has a metabolic potential comparable to the liver^10,11^, and thus, a relevant study addressing the complex liver/gut interconnection could be useful for the prevention and therapy of gut dysbiosis, ameliorating many complications in LC. The pioneering work of Li^12^ introduced the term ‘functional metagenomics’, intended as a multivariate statistical tool to discover functional relationships among bacterial species and metabolites derived from bacteria/host cometabolism: very few studies have attempted such an integrated approach in clinical research^13–15^. In LC, the combination of metagenomics (or 16S-targeted sequencing) and metabolomics would give insights into the yet unknown role of bacterial species involved in BT and liver functionality, especially when considering portal blood as the main route for the gut-liver axis^16,6,17^. The influence of the gut microbiota on host health is an increasingly concept progressively accepted, but the connection between gut microbiota BT and LC remains to be investigated. To outline the impact of the gut microbiota on LC and its physiopathological implications, we used the integration of omics platforms^18^ to describe the microbial compositional shifts in caecal biopsies, faeces, and peripheral and portal blood from LC patients, relating them to clinical parameters.

## Results

### Microbiota characterization of biopsies, faeces, and blood samples

**Figure 1:**
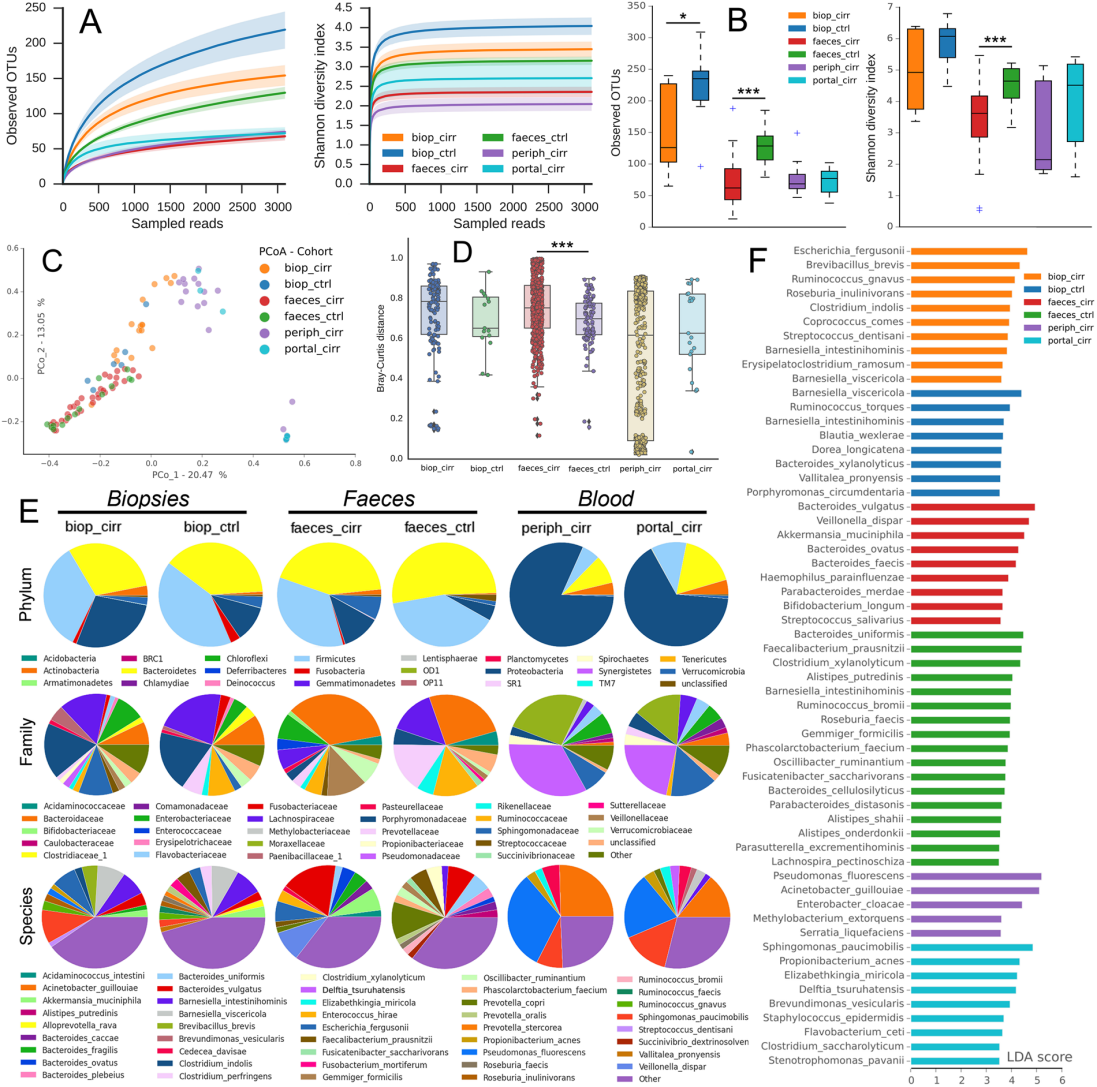
Microbiota compositional analysis. Average rarefaction curves (with 95% confidence interval) (panel A) and box plots (panel B) of α-diversity richness (observed OTUs) and biodiversity (Shannon index) estimators are reported for each dataset. The PCoA analysis (for β diversity) was based on the Yue & Clayton measure of dissimilarity (panel C), while the within-group Bray-Curtis average distance is reported in (panel D). Pairwise comparisons (panel E) were performed on the average relative abundances at the phylum, family (only ≥ 0.5%) and species (only ≥ 0.5%) levels for all six datasets (biop_cirr, biop_ctrl, feces_cirr, feces_ctrl, periph_cirr, portal_cirr) and reported as pie charts. All families or species whose mean relative abundance is < 0.5% collectively fall within the ‘Other’ group. Lefse analysis (panel F) was performed on all bacterial species, reporting the most discriminant ones (LDA score > 3.5) in decreasing order for each dataset. *P* values: *≤0.05, **≤0.01, ***≤0.001.

We used 16S rRNA V3-V4 targeted sequencing to characterize the microbiota composition of 89 samples from cirrhotic patients (17 caecum, 35 faeces, 30 peripheral blood, 7 portal blood) and 20 samples from controls (6 caecum, 14 faeces). We obtained a total of 9209053 filter-quality reads (84487 per sample on average), which were clustered into a total of 1990 OTUs (see Supplementary Fig. S1, Supplementary Table S2) and classified with the NCBI database. Estimates of the richness (observed OTUs) and biodiversity (Shannon) evidenced a marked dysbiotic status in cirrhotic faeces, with an α diversity similar to that of blood samples (Fig. 1A,B). PCoA analysis (for β diversity) computed on the relative abundances of OTUs showed a significant separation among the samples classified by their origin (biopsies, faeces, blood) (AMOVA *P* < 0.001, HOMOVA *P* < 0.001, Bonferroni pair-wise error rate: 0.0033, RF error rate: 0.266) (Fig. 1C). No significant separation of samples was obtained after classification by aetiology or drug usage (Supplementary Figs S2, S3 and S4). In particular, cirrhotic patients’ faecal microbiota structure was different from that of the controls (*P* < 0.001) and also had a higher within-group Bray-Curtis distance (Fig. 1D) (0.75 ± 0.006 vs 0.69 ± 0.01, *P* = 5.14*10^−5^). Pairwise statistical analyses (Fig. 1E, see supplementary file) showed, at the phylum level, a greater proportion of Verrucomicrobia (+4.1-fold, *P* = 0.013) and Fusobacteria (*P* = 0.005) in cirrhotic faeces and a greater proportion of Proteobacteria in cirrhotic biopsies, although the latter was not significant (+1.5-fold, *P* = 0.074) (see Supplementary Table S3). Surprisingly, Proteobacteria were predominant in both peripheral and portal blood (81.2% vs 65.1%, *P* = 0.43) and present in a higher proportion in the portal blood of extremophiles or uncultured phyla. Pairwise analysis also revealed 52 and 107 species with significant differences between cirrhotic patients and controls in the biopsies and faeces, respectively, while 71 species differed significantly between the peripheral and portal blood of cirrhotic patients (Fig. 1E, see Supplementary Table S4, supplementary file). To facilitate the analysis, the Lefse algorithm was implemented to determine all discriminant bacterial species having an LDA score higher than 3.5 (Fig. 1F): the most significant were *Escherichia fergusonii* for biop_cirr, *Barnesiella viscericola* for biop_ctrl, *Bacteroides vulgatus* for feces_cirr, *Bacteroides uniformis* for feces_ctrl, *Pseudomonas fluorescens* for periph_cirr and *Sphingomonas paucimobilis* for portal_cirr. Overall, these results revealed a marked dysbiotic status in cirrhotic patients’ faeces and biopsies: at the same time, they highlighted the importance of Proteobacteria in cirrhotic patients’ peripheral and portal blood.

### Cirrhotic patients show faecal alterations mainly in SCFA, carbon and methane metabolism

**Figure 2:**
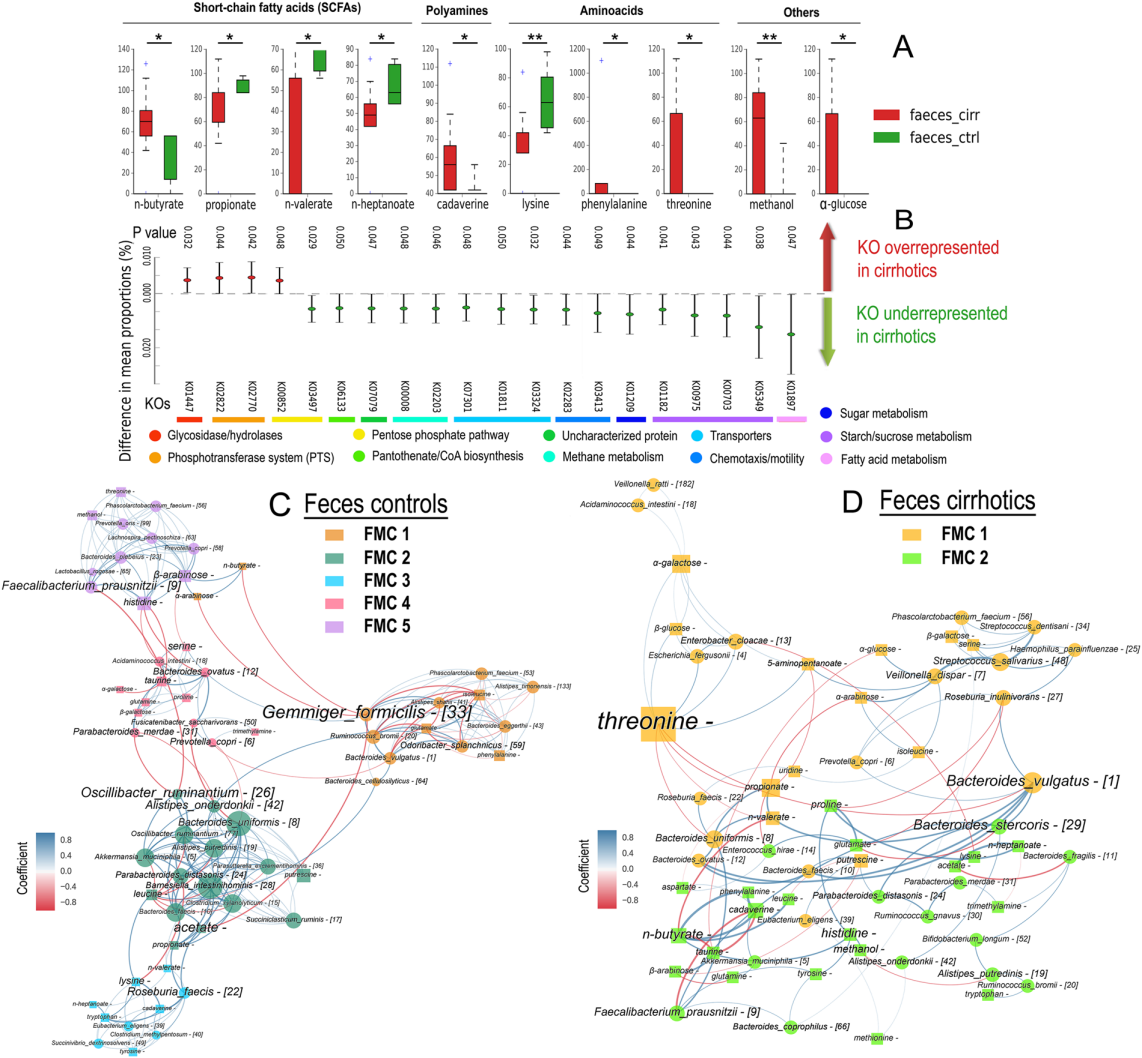
Faecal metabolomics and network analysis. The Mann-Whitney U test was employed to assess putative differences among faecal metabolites (from NMR) of cirrhotic patients (red) and controls (green) (panel A). Scaled values on the y-axis are arbitrary units referring to peak area. *P* values: *≤0.05, **≤0.01. PICRUSt analysis (panel B) was employed to predict metagenomes from the 16S data and to infer differences in mean proportions (expressed as %) among cirrhotic patients (red) and controls (green), for the first 20 Kegg Orthologues (KOrths) ordered by decreasing effect size (η^2^). The specific mean contributions of bacterial phyla and genera to the 10 KOrths overrepresented and to the 8 KOrths underrepresented in cirrhotic patients were calculated with PICRUSt (see supplementary Fig. S4). Co-occurrence network analysis was performed on faecal 16S and NMR merged datasets for both controls (panel C) and cirrhotic patients (panel D). The Pearson coefficient (*r*), ranging from positive (blue) to negative (red) values, is reported (edges with −0.7 > *r* > 0.7), based on correlation heatmaps (see supplementary Fig. S5). The edge thickness is proportional to the number of co-occurrences found between two nodes (species or metabolites) linked by the edge itself. Bacterial species having a mean relative abundance ≥0.5% were reported with their OTU number (squared brackets) and represented as circles, while metabolites were represented as squares within networks. Node size is proportional to the number of edges departing from the node, indicating its degree of interaction. Node name size is proportional to the betweenness centrality, meaning the bridging/key importance of that node within the network. Nodes are coloured by modularity class (community detection algorithm) to identify different functional metagenomic communities (FMCs) for the controls (5 FMCs) and cirrhotic patients (2 FMCs).

Upon finding a dysbiotic faecal microbiota composition in cirrhotic patients, we sought to investigate putative differences in faecal metabolite profiles. The NMR profiles of cirrhotic patients were significantly different from those of the controls (χ^2^ = 10.41, *P* = 0.0013), exhibiting altered levels of amino acids, SCFAs, methanol, cadaverine and α-glucose (Fig. 2A). To assess the metabolic potential of faecal microbial communities, PICRUSt analysis was implemented on the faecal 16S data to infer gene KEGG orthologue (KOrth) abundances, pathways involved (ko), and phylum/genus contributions (see Supplementary Fig. S5). Upon comparing the faeces of cirrhotic patients and controls, we retrieved 432 significantly different KOrths in a total of 6909, while 8 pathways were underrepresented and two overrepresented in cirrhotic patients (Fig. 2B). Cirrhotic patients showed 10 enhanced KOrths, regarding sugar-related transport (fructose, ascorbate), DNA repair, and defence against oxidative stress and toxins (Fig. 2B, see Supplementary Table S5). Taking into account the significant NMR metabolites retrieved by pairwise statistics, we found 31 KOrths reduced in cirrhotic patients (Fig. 2B, see Supplementary Table S6), especially those for SCFA (ko00650 butanoate, ko00640 propanoate), carbon (ko01200) and methane (ko00680) metabolism. While the overrepresented pathways were more heterogeneous, the underrepresented pathways were strongly dominated by carbon metabolism (17/31 KOrths, ~55%) and methane metabolism (11/31 KOrths, ~35.5%), with some KOrths involved in other pathways (e.g., ko00010 glycolysis/gluconeogenesis).

### Functional metagenomic networks (FMNs) reveal a ‘functional dysbiosis’ in cirrhotic patients

After finding that dysbiosis in cirrhotic patients faeces was accompanied by alterations in metabolites, we combined 16S sequencing, NMR metabolomics and network analysis, resulting in functional metagenomics networks (FMNs). The control network showed a discrete clustering of bacterial species or metabolites, showing five definite functional metagenomics communities (FMCs), while the cirrhotic network was sparser, with only two FMCs (Fig. 2C,D, Supplementary Table S7). *Gemmiger formicilis*, *Oscillibacter ruminantium*, *Roseburia faecis* and *Faecalibacterium prausnitzii* were keystone species within the control network (Fig. 2C, Table 1). These species, all members of the Firmicutes phylum, are significantly higher in the controls than in cirrhotic patients (*Gemmiger formicilis*, 2.23% vs 0.82% *P* = 0.0082; *Oscillibacter ruminantium*, 2.95% vs 0.72% *P* = 2.6^*^10^−5^; *Roseburia faecis*, 1.73% vs 0.81% *P* = 0.0081; *Faecalibacterium prausnitzii*, 5.47% vs 1.76% *P* = 0.0047, see supplementary file), and Lefse analysis showed their importance in the controls (Fig. 1F). Due to their involvement in the production of SCFAs (especially butyrate), they could exert a homeostatic role within the intestine of the controls^19,20^: here, *Gemmiger formicilis* is the most important keystone species^21^. The cirrhotic FMN had different properties from the controls: i) a lower number of edges; ii) a lower percentage of synergistic interactions (blue edges); iii) a lower synergistic/competitive ratio; and iv) lower density and modularity (Table 1). Notably, threonine had the highest importance within the cirrhotic network (was a keystone metabolite), having the highest betweenness centrality (Table 1, Fig. 2D). Threonine was positively linked (blue edges) with *Escherichia fergusonii* (Otu4) and *Enterobacter cloacae* (Otu13) but negatively related (red edges) to *Bacteroides vulgatus* (Otu1) and *Bacteroides uniformis* (Otu8). Thus, an increase in Otu4 or Otu13 or a decrease in Otu1 or Otu8 was expected to increase threonine levels, as observed in the NMR results (Fig. 2A). Interestingly, the levels of *Escherichia fergusonii* and *Enterobacter cloacae* were higher in cirrhotic patients than in the controls (6.63% vs 0.04% *P* = 0.003, and 1.56% vs 0.00% *P* = 0.003, respectively), while the level of *Bacteroides vulgatus* was lower (8.57% vs 16.98% *P* = 0.048) (see Supplementary Table S4, supplementary file). Overall, the FMN results highlighted a ‘functional dysbiosis’ in cirrhotic faeces compared to control faeces, with specific keystone features.

**Table 1:** Descriptive parameters of faecal functional metagenomics networks (FMNs).

Parameter	FMN Controls (n = 14)	FMN Cirrhotic Patients (n = 35)
Nodes	63	62
Edges	263	112
Synergistic interactions (%)	223 (84.8)	88 (78.6)
Competitive interactions (%)	40 (15.2)	24 (21.4)
Syn/Com ratio	5.58	3.67
Density	0.135	0.059
Modularity	0.589	0.401
Keystone species (BC, FMC, [Otu], Rel.abund.%)	*G. formicilis* (442.4, I, [33], 2.23) *O. ruminantium* (289.4, II, [26], 2.95) *F. prausnitzii* (231.1, V, [9], 5.47) *A. onderdonkii* (204.8, II, [42], 0.72) *R. faecis* (194.9, III, [22], 1.73)	*B. vulgatus* (277.1, I, [1], 16.98) *B. stercoris* (264.7, II, [29], 1.18) *F. prausnitzii* (209.6, II, [9], 1.76) *V. dispar* (174.4, I, [7], 9.68) *B. uniformis* (171.2, I, [8], 2.06)
Keystone metabolites (BC, FMC)	acetate (279.3, II) serine (162.0, IV) β-arabinose (158.3, V) lysine (147.4, III) histidine (121.7, V)	threonine (332.5, I) n-butyrate (256.2, II) histidine (242.9, II) methanol (197.7, II) proline (176.8, II)

BC = betweenness centrality value.

FMC = functional metagenomics community (in Roman numbers).

Otu = operational Taxonomical Unit.

Rel.abund. = relative abundance (%).

### Proteobacteria and iron metabolism are functionally linked within cirrhotic patients’ blood microbiota

**Figure 3:**
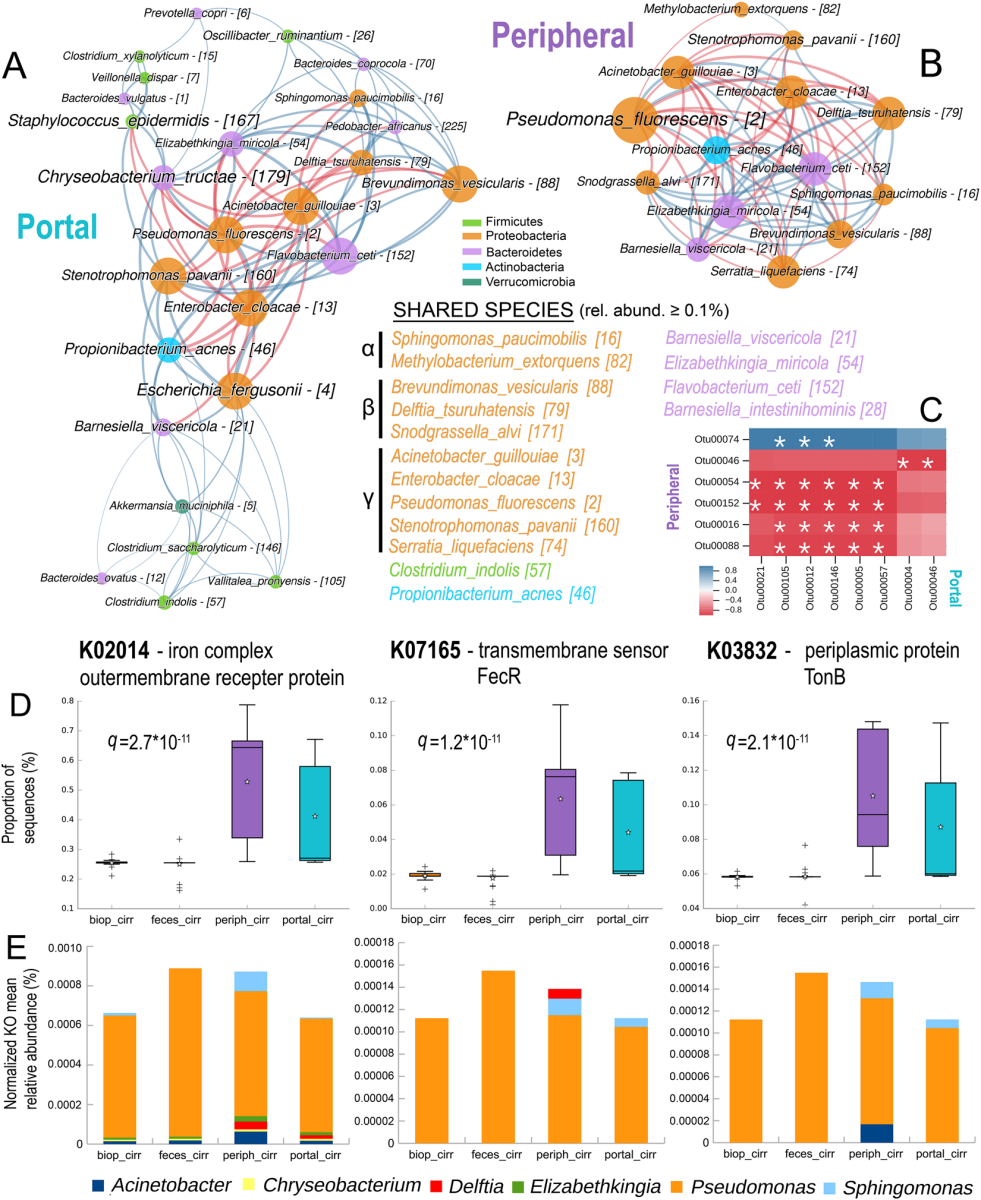
Network and PICRUSt analysis of peripheral and portal blood microbiota in cirrhotic patients. Network analysis was performed on portal (panel A) and peripheral (panel B) blood, taking into account OTUs (within square brackets) with mean relative abundance ≥0.5%. Network properties as in Fig. 2. The crosscorrelation heatmap (panel C) depicts interrelationships and was built using Pearson coefficients, with a white star indicating a significant correlation (*P* < 0.05 after FDR correction). PICRUSt analysis (panel D) shows differences, among the four cirrhotic patient datasets, in the sequence numbers (expressed as % of the total) relative to three KOrths involved in bacterial iron transport: the peripheral and portal blood are significantly enriched in these three genes (Kruskal-Wallis test, Benjamini-Hochberg FDR *q*-value). Within boxes, stars represent the mean, while horizontal bars represent the median. Species percentage contributions to the three iron-related KOrths (panel E) were computed with PICRUSt and are reported as ‘normalized KO mean relative abundance (%)’ on the y-axis: normalization was performed according to the total number of sequences and number of samples for each cohort (biop_cirr, feces_cirr, periph_cirr, portal_cirr).

In cirrhotic patients, we observed differences between the peripheral and portal blood microbiota (Fig. 1, see supplementary file). Then, network analysis was employed to infer functional inter- and intra-relationships. As found with pairwise analysis (Fig. 1E), the ‘liquid biopsy’ showed a great prevalence of Proteobacteria members in both environments: 10/14 (71.4%) and 8/26 (30.8%) nodes in the peripheral and portal blood networks, respectively, belonged to this phylum, (Fig. 3A,B). Sixteen species (with mean relative abundance ≥0.1%), mainly belonging to the Proteobacteria phylum (10/16, 62.5%), were found to be common to the peripheral and portal blood (Fig. 3), suggesting their possible role as hepatic barrier crossers in liver cirrhosis. Via correlation analysis, we found a total of 27 significant interrelationships: 3/27 (11.1%) were positive, while 24/27 (88.9%) were negative (intercompetition) (Fig. 3C). All Proteobacteria members had the highest degree of interaction (intrarelationships) within both networks (represented by node size), and in the peripheral blood, they also acted as keystones species (*Pseudomonas fluorescens, Stenotrophomonas pavanii*, *Acinetobacter guillouiae*) (Table 2), indicating a functional role within this blood environment. In this view, the peripheral network was more compact (density close to 1, modularity close to zero), with competitive interactions outnumbering synergistic ones (Table 2). Thus, it seems that a stable and definite peripheral blood microbiota was present in cirrhotic patients. The peripheral network also had the lowest syn/com ratio (*P* = 0.003, χ^2^ = 8.85), with the majority of retrieved Proteobacteria species showing only negative edges among themselves (intracompetition). Interestingly, most of these species are strong producers of iron chelators (siderophores)^22–24^, and PICRUSt analysis showed that both the peripheral blood and the portal blood of cirrhotic patients were significantly enriched in bacterial KO genes linked to active iron transport (Fig. 3D). After normalization for gene copies and number of samples, PICRUSt showed great contributions of the *Pseudomonas*, *Sphingomonas*, *Acinetobacter* and *Delftia* genera in the peripheral and portal blood to iron-related KOrths (K02014, K07165, K03832) (Fig. 3E), while only *Pseudomonas* contributed greatly to the same KOrths in cirrhotic biopsies or faeces. Hence, even if intra- and intercompetitive interactions (red edges) dominated within Proteobacteria, the higher diversity in KOrth contributions seen within the peripheral blood could be an advantage enabling iron-demanding species to thrive in this harsh environment^22,25^.

**Table 2:** Descriptive parameters of peripheral and portal networks.

Parameter	Peripheral (n = 30)	Portal (n = 7)
Nodes	14	26
Edges	73	108
Synergistic interactions (%)	36 (49.3)	78 (72.2)
Competitive interactions (%)	37 (50.7)	30 (27.8)
Syn/Com ratio	0.97	2.60
Density	0.802	0.332
Modularity	0.033	0.319
Keystone species (BC, Otu, Rel.abund.)	*P. fluorescens* (8.94, [2], 31.97) *S. pavanii* (3.00, [160], 0.53) *A. guillouiae* (0.94, [3], 25.91)	*C. tructae* (46.2, [179], 0.51) *E. fergusonii* (42.5, [4], 1.28) *S. epidermidis* (37.1, [167], 0.88)

BC = betweenness centrality value.

Otu = operational taxonomical unit.

Rel.abund. = relative abundance (%).

### Bacterial species, genes and metabolites related to hepatic encephalopathy

After assessing the compositional shifts of faeces and blood in cirrhotic patients, we sought to find species, genes and metabolites eventually involved in HE. Among the bacterial species able to translocate across the hepatic barrier (Fig. 3), we found that *Stenotrophomonas pavanii* (rel.abund. = 0.53%, a keystone species, see Table 2) and *Methylobacterium extorquens* (rel.abund. = 1.13%) in the peripheral blood raised the risk of HE by 135% and 142%, respectively, while *Clostridium indolis* lowered it by 10%, thus performing a protective role (Table 3). Within the faeces, *Bacteroides coprocola* (+131%) and *Bifidobacterium longum* (+113%) enhanced the risk of HE, while *Bacteroides faecis* and *Bacteroides coprophilus* lowered it by 43% and 34% (Table 3). The gene *msrP*/K07147 (a methionine sulfoxide reductase) within the peripheral blood was uniquely related to a higher risk of HE (+37%), while within the faeces, the genes *pdhD*/K00382 (+38%), *sugE*/K11741 (+36%), and *ssb*/K03111 (+45%) were related to a higher risk of HE (Table 3). Interestingly, the gene *tonB*/K03832 (a periplasmic protein involved in iron metabolism) within the faeces was protective against HE (−29%). Three keystone faecal metabolites (see Table 1) were related to HE: methanol (+71%) and threonine (+38%) heightened the risk, while n-butyrate (−23%) lowered it. Peripheral blood IL6 was also positively related to HE (+28%).

**Table 3:** Microbiota features related to hepatic encephalopathy (HE) in cirrhotic patients.

Species, KOrths, Metabolites	Logistic Reg. Coeff.	Odds Ratio (OR)	Randomized Lasso Coeff. (RLC)	Elastic Net Coeff. (ENC)	SGDC Coeff.
**Peripheral blood**					
*Clostridium indolis*	−0.151	0.90	0.530	−0.049	−0.316
*Methylobacterium extorquens*	1.275	2.42	0.445	0.042	0.642
*Stenotrophomonas pavanii*	1.231	2.35	0.555	0.082	0.665
K07147	0.450	1.37	0.205	0.033	0.562
IL6	0.355	1.28	0.800	0.057	0.319
**Faeces**					
*Bacteroides coprocola*	1.210	2.31	0.745	0.051	0.738
*Bacteroides coprophilus*	−0.609	0.66	0.890	−0.005	−0.371
*Bacteroides faecis*	−0.804	0.57	0.785	−0.033	−0.465
*Bifidobacterium longum*	1.092	2.13	0.790	0.096	0.728
K00382	0.466	1.38	0.305	0.046	0.729
K11741	0.446	1.36	0.190	0.020	0.695
K03111	0.540	1.45	0.175	0.023	0.664
K03832	−0.496	0.71	0.100	−0.012	−0.663
methanol	0.772	1.71	0.260	0.136	0.697
threonine	0.466	1.38	0.005	0.007	0.500
n-butyrate	−0.371	0.77	0.005	−0.018	−0.340

K07147: methionine sulfoxide reductase catalytic subunit *msrP* [EC:1.8.-.-].

K00382: dihydrolipoamide dehydrogenase *pdhD* [EC:1.8.1.4].

K11741: quaternary ammonium compound-resistance protein *sugE*.

K03111: single-strand DNA-binding protein *ssb*.

K03832: periplasmic protein *tonB*.

### Correlation of clinical parameters with bacterial species and metabolites in cirrhotic patients

**Figure 4:**
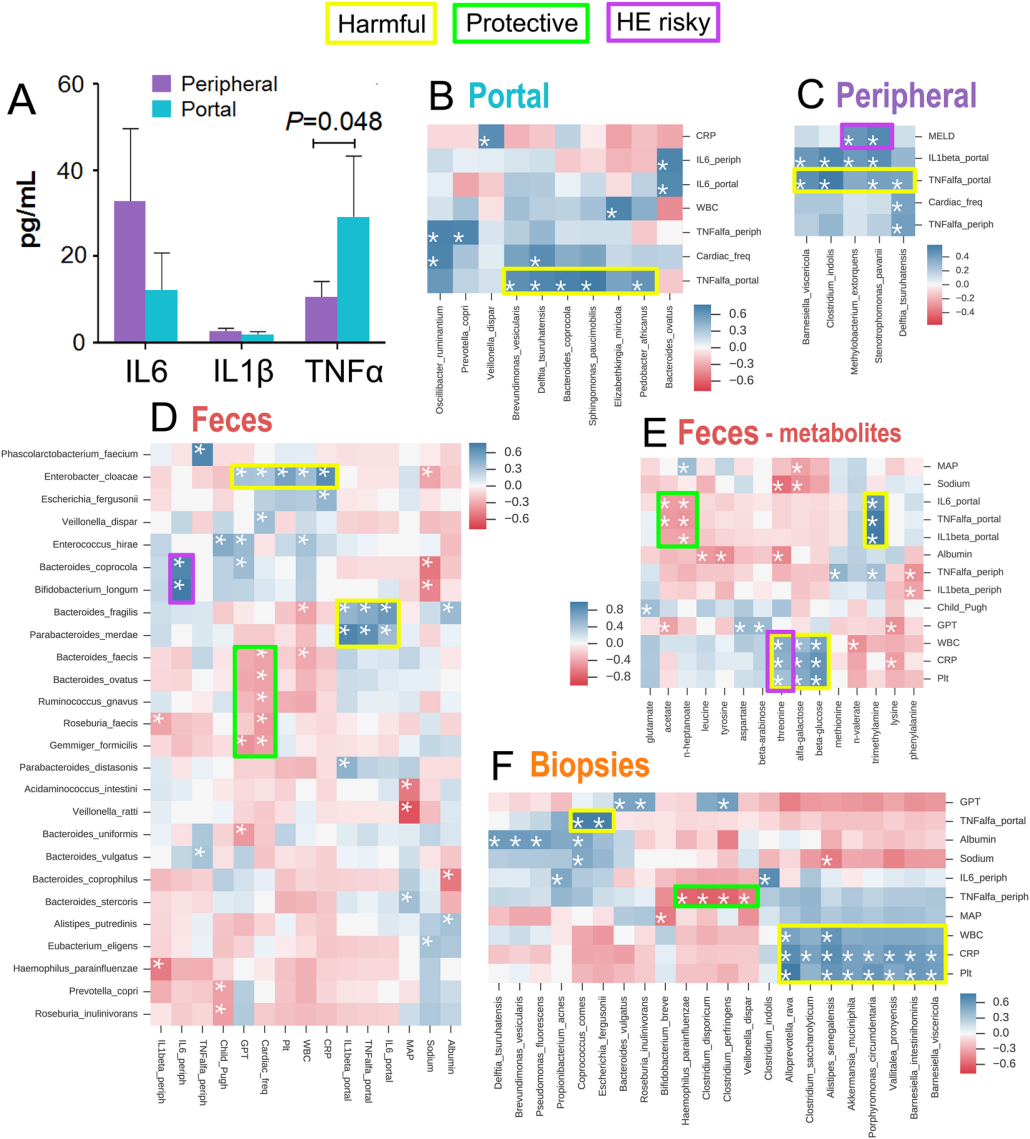
Cross-correlation of metagenomics/metabolomics datasets with clinical parameters in cirrhotic patients. ELISA tests for proinflammatory cytokines were performed on peripheral and portal blood (panel A). The Pearson coefficient (*r*), ranging from positive (blue) and negative (red) values, was used to cross-correlate bacterial species (within portal/peripheral blood, biopsies, faeces) and clinical parameters (ELISA included) for cirrhotic patients (see Supplementary Table S1) (panels B–F). A white star indicates a significant correlation (*P* ≤ 0.05 after FDR correction). Rectangles denote harmful (yellow), protective (green), or high-HE-risk (purple) consortia/metabolites.

We measured three proinflammatory cytokines (IL6, IL1β, TNFα) in peripheral and portal blood samples (Fig. 4A). TNFα was significantly higher in the portal blood than in the peripheral blood (29.1 ± 14.4 pg/mL *vs* 10.5 ± 3.6 pg/mL, *P* = 0.048), and through Pearson correlation, we found portal TNFα to be positively related to specific bacterial consortia within the portal, peripheral, faecal, and intestinal habitats (Fig. 4). *Delftia tsuruhatensis*, one of the 16 species shared between peripheral and portal blood, was positively linked to portal TNFα and cardiac frequency when present within both portal (rel.abund. = 3.04%, *r* = 0.86/*P* = 0.013, *r* = 0.80/*P* = 0.029) and peripheral blood (rel.abund. = 1.37%, *r* = 0.39/*P* = 0.035, *r* = 0.39/*P* = 0.034) (Figs. 4C,D). Interestingly, the high-HE-risk consortium *Methylobacterium extorquens*/*Stenotrophomonas pavanii* in the peripheral blood was positively correlated with the MELD score (*r*_mean_ = 0.48/*P*_mean_ = 9.6^*^10^−3^) and portal proinflammatory cytokines (TNFα *r* = 0.40/*P* = 0.03; IL1β *r* = 0.49/*P* = 0.007) (Fig. 4C). The high-HE-risky consortium *Bacteroides coprocola/Bifidobacterium longum* in the faeces was strongly correlated with peripheral IL6 (*r*_mean_ = 0.81/*P*_mean_ = 3.5^*^10^−8^) (Fig. 4D). Within cirrhotic patients’ faeces (Fig. 4D), *Enterobacter cloacae* (rel.abund. = 1.56%) was positively correlated with five parameters (*r*_mean_ = 0.49/*P*_mean_ = 0.019): GPT, cardiac frequency, platelets, WBC, and CRP. *Escherichia fergusonii*, another Proteobacteria member that is closer (blue edge) to *Enterobacter cloacae* within cirrhotic FMC1 (Fig. 2D), gave a significant result only with CRP (*r* = 0.46/*P* = 0.006), although its correlation pattern is similar to that of *E. cloacae*. Five faecal species, *Gemmiger formicilis*, *Roseburia faecis*, *Ruminococcus gnavus*, *Bacteroides ovatus* and *Bacteroides faecis*, were negatively correlated with cardiac frequency (*r*_mean_ = −0.37/*P*_mean_ = 0.031). Two faecal Bacteroidetes members, *Bacteroides fragilis* and *Parabacteroides merdae*, showed a positive correlation with portal blood proinflammatory cytokines (IL6, TNFα, IL1β) (*r*_mean_ = 0.59/*P*_mean_ = 0.004). Another Bacteroides member, *Prevotella copri*, had a significantly higher relative abundance in portal blood than in peripheral blood (*P* = 0.0308) and was positively correlated with peripheral TNFα (Fig. 4B). Regarding faecal metabolites (Fig. 4E), the harmful trimethylamine^26^ had a strong positive correlation with the portal blood proinflammatory cytokines IL6, TNFα and IL1β (*r*_mean_ = 0.92/*P*_mean_ = 3.7^*^10^−6^), while two SCFAs, acetate and n-heptanoate, were negatively correlated with these cytokines (*r*_mean_ = −0.46/*P*_mean_ = 0.037), thus performing a protective role. Threonine (a key metabolite within cirrhotic FMN, Fig. 2D, Table 1), α-galactose and β-glucose were positively correlated with WBC, PCR and platelets counts (*r*_mean_ = 0.64/*P*_mean_ = 0.009). Overall, we showed that some of the sixteen species shared among portal and peripheral blood were significantly correlated with systemic inflammation, HE and worsening of clinical and biochemical parameters in cirrhotic patients.

## Discussion

The hepatic portal system, collecting gut microbiota metabolites and byproducts, could act as a highway for bacterial translocation, leading to physiopathological complications in liver cirrhosis, such as hepatic encephalopathy. A combination of 16S targeted sequencing and metabolomics was employed on LC biopsies, faeces and portal/peripheral blood to extrapolate species, genes (KEGG Orthologues, KOrths) and faecal metabolites able to impact liver functionality in a feedback loop through the bloodstream. We found that cirrhotic patients’ portal blood had a bacterial community composition similar to that of the colonic mucosa, but not to that of the faeces. These findings suggest that BT derives primarily from mucosa-associated species, rather than luminal ones, as previously suggested^6,27^. As previously reported^28^, the blood microbiota could be completely derived from a leaky intestinal barrier^27,29^, strengthening the importance of mucosa-associated microbiota in BT. A reduced secretion of bile acid production in cirrhosis has been reported, favouring the overgrowth of pathogenic and proinflammatory members of the microbiome including Porphyromonadaceae and Enterobacteriaceae^30,31^. The relative scarcity of secondary biliary acids significantly correlates with the reduction in the Clostridium cluster XIVa group; this correlation is probably due to the high proportion of 7α-dehydroxylating bacteria within this cluster. In animal models, the production of secondary bile acids by this group of bacteria causes positive regulation of bile acid synthesis in the liver. In fact, a higher concentration of secondary bile acids in the ileum implies a lower concentration of tauro-β-muricholic acid, which is an inhibitor of hepatic bile acid synthesis (via the inhibition of FXR signalling)^32^. In this “liver-gut axis perspective”, as the severity of cirrhosis progresses, lower amounts of secondary bile acids reach the large bowel: in particular, deoxycholic acid (DCA) is the one that displays the most potent antimicrobial activity^33^. Thus, the consequence of its reduced concentration is a higher risk of bacterial overgrowth in the small bowel, often characterized by reduced biodiversity^34^. The interplay among intestinal permeability, compositional shifts in mucosa-associated microbiota and BT is thus of great importance in diseases affecting the liver, such cirrhosis, NAFLD, NASH, and, ultimately, liver cancer^27^. We found sixteen species shared between the portal and peripheral blood in cirrhotic patients (Fig. 3). A caveat of our study would be the use of bacterial DNA (bDNA) as a BT marker, which raises the question of the role of resident bacteria in both portal and peripheral blood^35^. Even if a fraction of bDNA could derive from the recently proposed ‘relic DNA’^36^, it is debatable whether it would affect biodiversity measures^37^, especially within the blood habitat, where bacterial species usually survive at low levels due to iron depletion^25,38^. It seems that Proteobacteria members are the major constituent in the peripheral blood, especially in a diseased state^39^, which highlights their role in bacterial translocation and suggests that elevated LPS and bDNA loads could exacerbate a systemic immune activation^40^. Our network analysis showed that the main bacterial translocators were *Pseudomonas* and other Proteobacteria members (mainly αβγ-proteobacteriaceae). These keystones and shared species (Fig. 3) are potential or well-recognized pathogens living in competition with each other and with the host for scarce resources such as iron availability. Interestingly, PICRUSt analysis showed that both the peripheral blood and the portal blood of cirrhotic patients were significantly enriched in bacterial KOrth genes linked to active iron transport (Fig. 3D). Ultimately, all these Proteobacteria species are strong producers of siderophores^23–25^ and are able to outcompete vertebrates in iron scavenging^25,38^. Due to the higher abundance of iron transport-related bacterial genes in the peripheral and portal blood, iron supplementation could allow Proteobacteriaceae members, which usually harbour genotoxins, to flourish^24,41^. Excessive iron has well-recognized hepatotoxic activity and is able to interfere with interferon therapy and to induce oxidative stress (free radicals)^42^. Moreover, excessive iron is linked to liver fibrosis, DNA damage, enhanced predisposition to gut/liver cancer^43^, and especially the overgrowth and virulence of bacterial pathogens^24^. In IBD, it was found that iron supplementation was related to colitis severity^44^, while replacement therapy was related to a diminution of beneficial Clostridiales and lesser biodiversity^45^. We revealed a link between the worsening of clinical parameters in LC and species able to translocate through the gut barrier, especially gram-negative members of the Proteobacteria phylum. Gram-negative-derived LPS interacts with macrophages, releasing proinflammatory cytokines such as TNF-alpha, IL-6, and IL-1b^46^. We found higher levels of TNFα within the portal blood, which could support the role of the portal blood microbiota in inducing a “cytokine storm”, exacerbating liver failure and clinical symptoms^46,47^. Interestingly, we found specific bacterial consortia and metabolites to be involved in worsening systemic inflammation. Within the Bacteroides phylum, the species *Prevotella copri*, previously linked to rheumatoid arthritis^48^ and stage 4-HCV patients^49,50^, was positively correlated with higher levels of peripheral TNFα when present in the portal blood. *Prevotella copri* was higher in cirrhotic patients’ biopsies and faeces, though not significantly, and this abundance could be explained by its role as an enterotype linked to carbohydrate metabolism^49^, which we found to be altered in LC. In this respect, our results are in accordance with two recently published papers^51,52^, although some differences exist in the levels of SCFAs (especially butyrate), perhaps due to geographical constraints or cohort size. The presence of Proteobacteria members within the portal and peripheral blood was positively linked to IL6, IL1β, TNFα, GPT and cardiac frequency. Faecal trimethylamine (TMA) had a strong positive correlation with portal blood proinflammatory cytokines IL6, TNFα and IL1β. High levels of TMA in the faeces are generated by the gut microbiota^53^, probably leading to altered levels of the toxic compound trimethylamine *N*-oxide (TMAO) within the liver and affecting its functionality^26^. Thus, higher levels of TMAO and TNFa/IL6/IL1b levels could lead to a synergistic feedback loop in liver failure. Faecal acetate and n-heptanoate were negatively related to portal blood proinflammatory cytokines, indicating a protective role. Eight out of 46 cirrhotic patients in our series had HE at hospitalization (see Supplementary Table S1), and the HE risk was raised by *Methylobacterium extorquens* and *Stenotrophomonas pavanii* in the peripheral blood. Interestingly, these two species were positively correlated with one another (Fig. 3B, blue edge) and with the MELD score (Fig. 4C), emphasizing their interplay with severe liver failure and HE. The species most strongly related to HE (OR = 2.42, RLC = 0.445) was *Methylobacterium extorquens*, an opportunistic pathogen able to i) oxidize methanol to formaldehyde (a compound responsible for chronic solvent-induced encephalopathy - CSE)^54^ and ii) live in the peripheral blood, thus possibly causing systemic disease^55^. Interestingly, faecal methanol (a keystone metabolite) was positively related to HE (Table 3) and was significantly higher in LC (Fig. 2A), suggesting a possible reservoir for *Methylobacterium extorquens* toxic activity within the peripheral blood. Peripheral IL6 was positively related to HE risk (Table 3) and to the faecal *Bacteroides coprocola*/*Bifidobacterium longum* consortium (Fig. 4D): importantly, these two species also enhanced HE risk (Table 3). Our results confirmed the predictive role of IL6 in HE^56^ but, surprisingly, are contrary to previous observations on *Bifidobacterium longum*. In fact, this species has been utilized to treat minimal hepatic encephalopathy (MHE)^57^; thus, its increased risk for HE (OR = 2.13, RLC = 0.79) warrants more clinical and molecular studies on its use as a psychobiotic^58^. Interestingly, n-butyrate was protective against HE; thus, providing LC subjects with a strong butyrate producer such as *Faecalibacterium prausnitzii* (a keystone species, Table 1) would probably ameliorate their conditions, although this hypothesis deserves further evidence. Despite some potential improvements in patient number and NGS approach (shotgun), this study represents one of the first attempts at integrating metagenomic and metabolomic datasets to obtain a clinical meaning. We aimed to improve our knowledge of the gut-liver axis and to advance our ability to correct or prevent liver-related pathologies. Intervention by prebiotics/probiotics/synbiotics, diet or faecal microbiota transplant (FMT)^59^, along with cautious iron supplementation, could support the development of new customized treatments for LC patients with systemic inflammation and/or HE.

## Methods

### Subjects enrolled

All methods were carried out in accordance with relevant guidelines and regulations. Forty-six patients with liver cirrhosis (aged 60.3 ± 11.5 years, sex ratio M/F 32/13) hospitalized at the Department of Gastroenterology, University Hospital Policlinico Umberto I, were included in the study (see Supplementary Table S1). The diagnosis of cirrhosis was proven through liver biopsy or based on clinical, biochemical and ultrasonographic signs. The exclusion criteria were as follows: diagnosis of infection (based on fever, leukocytosis, elevated C Reactive protein (CRP), erythrosedimentation rate (ESR), procalcitonin, clinical symptoms, and positive microbiological cultures when present), use of systemic antibiotics in the last 3 months, variceal bleeding within the last 4 weeks, or alcohol or illicit drug intake within the last 3 months. Lactulose or rifaximin therapy was not considered cause for exclusion. No patient took other drugs that could potentially affect the microbiota (such as metformin). Patients with any type of immunodeficiency (HIV, immunosuppression) or with a diagnosis of hepatocellular carcinoma without Milano criteria were excluded. All the patients were followed throughout the time of hospitalization. Fourteen healthy age-matched individuals (aged 53.8 ± 7.8 years, sex ratio M/F 7/6) were recruited among their neighbours to serve as controls. Among this group, individuals who were taking, or had taken in the last 3 months, medications that could potentially modify the gut microbiota (antibiotics, probiotics) were excluded. The origin of cirrhosis, past and current complications of the disease, and laboratory findings (hemogram, serum electrolyte levels, renal and liver function tests, inflammatory parameters) were collected. The severity of liver disease was evaluated by the Child-Turcotte-Pugh (CTP) and model for end stage liver disease (MELD) scores. The chronic use of beta-blockers, lactulose, proton pump inhibitors (PPI) and other drugs with the potential to influence the gut microbiota was recorded. The patients included in the study and the healthy controls gave a fresh stool sample that was promptly stored at −80 °C. The cirrhotic patients also underwent serum collection of peripheral vein blood samples (2 mL) for cytokine titration (TNF-α, IL1β, IL6) and blood microbiota assessment. Portal blood (2 mL) was taken from seven cirrhotic patients admitted for TIPS procedures and was subjected to the same analyses as the peripheral blood. Both peripheral and portal blood samples were frozen at −80 °C immediately upon collection. For patients and controls undergoing a colonoscopy for the prevention of colorectal carcinoma, as indicated by clinical guidelines, or due to a general work up before being admitted to the liver transplant list, a mucosal biopsy (caecum) was obtained to assess the intestinal microbiota adhering to the mucosa. This biopsy was also immediately stored at −80 °C. All retrieved demographic and clinical parameters were anonymously used to build a matrix employed for subsequent multivariate statistical analysis.

### Ethical Statements

Both patients and controls signed an informed consent form, and the experimental protocol was approved by the Hospital ‘Umberto I – Policlinico di Roma’ Ethics Committee during the internal audit held on the 24^th^ of September 2015 (Protocol Number 2515/15, Rif. 3696) under the title ‘Microbiota composition in patients with hepatic cirrhosis’. The Ethics Committee operates under the standards of good clinical practice (GCP-ICH) and following the clinical duties of the Italian Ministry (D.M. 15/7/97, D.M. 18/3/98, D.Lgs. 24/6/2003, D.M. 12/5/2006, D.M. 21/12/2007, D.M. 8/2/2013). At enrolment, a general physical examination and vital signs were recorded. All methods were carried out in accordance with the relevant guidelines and regulations.

### ELISA

The peripheral and portal blood levels of the cytokines IL-6, TNF-α, and IL1-β were evaluated by enzyme-linked immunosorbent assay (ELISA). Briefly, 2 ml of peripheral or portal blood was collected as specified above in a test tube with anticoagulant and centrifuged at 3000 rpm for 10 minutes. One hundred microliters of supernatant was used to evaluate the cytokine levels via enzyme immunoassays carried out with commercial kits (Human ELISA Ready-SET-Go!, cat# 88-7066-22 for IL-6, cat# 88-7346-22 for TNF-α, cat# 88-7261-22 for IL1-β, eBioscience, San Diego, CA, United States), and assays were performed in triplicate following the manufacturer’s instructions. Plates (96-well ELISA plate, Corning Costar 9018, included in the kit) were read at 450 nm (subtracting the 570 nm readings as a baseline), and the intensity measurements were analysed with online software (http://www.elisaanalysis.com/) to retrieve values expressed in pg/mL (a four-parameter logistics curve was used for standard curve interpolation). A matrix of ELISA data was generated for subsequent multivariate statistical analysis. The Mann-Whitney U test was used to assess significant comparisons (*P* ≤ 0.05).

### Microbiota characterization of biopsies and of blood and stool samples

The biopsies underwent a first wash (30S mid-speed vortex) with 0.016% dithioerythritol (DTT, cat#D0632, Sigma-Aldrich, Milan, Italy) in phosphate-buffered saline (PBS, cat#AU-L0615-500, Aurogene, Rome, Italy) to remove mucus and were then washed three more times with PBS. The total DNA from stool samples (200 mg each), from biopsies (15 mg each) and from peripheral/portal blood (200 μl each) was automatically extracted with a Maxwell® RSC Instrument (Promega, Wisconsin, USA, kit #AS1400). For all three sample types, the manufacturer’s protocol was modified by incubating the samples with proteinase K at 56 °C, followed by a 4 hour incubation at 37 °C with 2 mg/ml (final concentration) lysozyme (cat# L6876, Sigma-Aldrich, Milan, Italy) to ensure a proper disruption of gram-positive bacterial species. Next-generation sequencing (NGS) of 16S rRNA V3-V4 regions amplicons was thus carried out on a total of 109 samples divided as follows: i) caecum samples (17 HC, 6 controls); ii) stool samples (35 HC, 14 controls); and iii) blood samples (30 HC peripheral, 7 HC portal). The samples were subjected to robotic PCR execution, library preparation and sequencing according to the Illumina 16S metagenomics standardized operational workflow (16S Metagenomic Sequencing Library Preparation, Part # 15044223 Rev. B). Appropriate blanks (negative controls) and mock communities (positive control) were employed to assess bacterial contamination throughout the NGS workflow and sequencing error rate. Each 16S library was checked for size with an Agilent 2200 Tapestation (Agilent Technologies, Santa Clara, CA, United States) and quantified with a Qubit 2.0 fluorometer using the Qubit dsDNA HS Assay Kit (cat# Q32851, Thermo Fisher Scientific, MA, United States). Sequencing was performed at the Italian Institute of Technology (https://www.iit.it/it/centers/clns-sapienza) with an Illumina MiSeq platform, Reagent Kit v3 (cat# MS-102-3003, Illumina, San Diego, CA, United States), 2 × 300 paired ends, and 600 cycles. The raw FASTQ files were analysed with Mothur pipeline v.1.38.1^60^ for the quality check and filtering (sequencing errors, chimaerae) on a Workstation CELSIUS R940 (Fujitsu, Minato-ku Tokyo, Japan). Filtered reads (9209053 in total, 84487 per sample on average, see Supplementary Table S3) were clustered into operational taxonomical units (OTUs), after the elimination of singletons and doubletons, by *de novo* OTU picking at 97% pairwise identity using standardized parameters and the SILVA rDNA Database v.1.19^61^ for alignment. In all, 1990 OTUs were identified. Given the high heterogeneity of the six datasets (biop_cirr, biop_ctrl, feces_cirr, feces_ctrl, periph_blood, portal_blood) in terms of OTUs and filtered quality read numbers, all samples were normalized to the number of reads present in the least rich sample (3101 reads for a portal blood sample). Sample coverage was computed with Mothur and found to be equal to 99% on average for all samples (mean ±SDM, 99.1% ± 0.5%), indicating that the normalization procedure was suitable for subsequent analyses. The analysis of molecular variance^62^ (AMOVA, which represents the difference in datasets’ centroids), homogeneity of molecular variance (HOMOVA, representing the difference in datasets’ standard deviations), parsimony test, LEfSe^63^, and random forest (RF) error rate were computed with Mothur v.1.38.1.

### OTU species assignment and multivariate statistical analyses

Bioinformatic and statistical analyses on recognized OTUs were performed with Python v.2.7.11. The most representative and abundant read within each OTU (as shown in the previous step with Mothur v.1.38.1) was subjected to a nucleotide Blast using the National Center for Biotechnology Information (NCBI) Blast software (ncbi-blast-2.3.0) and the latest NCBI 16S Microbial Database (ftp://ftp.ncbi.nlm.nih.gov/blast/db/). The retrieved species (first 500 OTUs) had the following Blast parameters values (mean ±SDM) (see supplementary file): E-value (1.82*10^–85^ ± 3.59^*^10^−85^), total score (703.5 ± 103.6), percentage identity (94.9 ± 4.0) and mismatches (21.2 ± 16.3). A matrix of bacterial relative abundances was built at each taxon level (phylum, class, order, family, genus, species, OTUs) for subsequent multivariate statistical analyses. Measurements of α diversity (within sample diversity) such as observed_otus and the Shannon index were calculated at the OTU level using the SciKit-learn package v.0.4.1. Exploratory analysis of β-diversity (between-sample diversity) was calculated using the Yue & Clayton measure of dissimilarity (*θ*) calculated with Mothur and represented in Principal Coordinate Analyses (PCoA), while ‘Bray-Curtis’ metrics and the ‘complete linkage’ method were used for hierarchical clusterization analysis (HCA) by implementing in-house scripts (Python v.2.7.11). To compare the microbiota taxa (at a mean relative abundance ≥ 0.5%) with demographic/clinical, NMR metabolomics, and ELISA datasets, a multivariate statistical Pearson correlation analysis (with related *P* values) was performed with in-house Python scripts. Pearson correlation matrices (metric = Bray-Curtis, method = complete linkage) were also generated for intra- and interdataset (biop_cirr, biop_ctrl, feces_cirr, feces_ctrl, periph_blood, portal_blood, NMR metabolomics) cluster generation and the discovery of positive/negative correlation coefficients. The Mann-Whitney U and Kruskal-Wallis tests were employed to assess significance for pairwise or multiple comparisons, respectively, considering a P value less than or equal to 0.05 to be significant. Cross-correlation Pearson matrices for network analysis (metric = Bray-Curtis, method = complete linkage) were generated with in-house scripts (Python v.2.7.11) and visualized with Gephi v.0.9.1^64^, considering OTUs having a mean relative abundance ≥ 0.5% and Pearson correlation coefficients −0.7 > *r* > 0.7, as previously reported^12^. A network analysis was performed on each dataset (biop_cirr, biop_ctrl, feces_cirr, feces_ctrl, periph_cirr, portal_cirr, NMR metabolomics, and merged 16S/NMR) using co-occurrences and visual representation as proposed by current guidelines^65–69^. The degree value, measuring the in/out number of edges linked to a node, and the betweenness centrality, measuring how often a node appears on the shortest paths between pairs of nodes in a network, were computed with Gephi v.0.9.1. Intranetwork communities (even for functional metagenomics communities, or FMCs) were retrieved using the Blondel community detection algorithm^70^ by means of randomized composition and edge weights, with a resolution equal to 1^71^. Clustering validation (K-means++^72^, Birch^73^, affinity propagation^74^) and performance measures (Silhouette score^75^, Calinski-Harabasz score^76^) were employed to confirm intranetwork clustering into communities. To find correlations among bacterial species, genes (KOrth, effect size ≥ 0.47), faecal metabolites and the presence/absence of hepatic encephalopathy (HE), we used logistic regression (−∞ < β < ∞), randomized lasso (0 < β < 1), elastic net (−∞ < β < ∞), and SGD classifier (−∞ < β < ∞) within the Python SciKit learn module^77,78^ on mean-centred and unit-variance dataframes. Odds ratios (ORs) were computed from logistic regression coefficients (β) with the formula OR = 2^β^^79^.

### Metagenome prediction and pathway analysis

A Biom file was generated with Mothur v.1.38.1 using the Greengenes database (v. 13_5_99) and used with PICRUSt 1.0.0 (Phylogenetic Investigation of Communities by Reconstruction of Unobserved States)^80^ with default parameters, in order to predict the Kyoto Encyclopedia of Genes and Genomes (KEGG) orthologues (KOrths) from 16S V3-V4 amplicon data. Specific KOrths related to significant NMR metabolites were bioinformatically assigned to each sample by means of the KEGG online website (http://www.genome.jp/kegg/ko.html) and Integrated Database Retrieval System (http://www.genome.jp/dbget/), taking into account the Orthology and Reactions databases for a refined search. STAMP^81^ was then utilized, employing the two-sided Welch’s t-test and η^2^ (effect size), to detect specific KOrths with discriminant power (*P* ≤ 0.05) and functional relationships to the NMR data on the cirrhotic/control faecal samples. The mean relative KOrth abundances, normalized by sample number, were computed with in-house Python scripts, and those lower than a definite threshold (2*10^−6^% for phylum, 5*10^−6^% for genus) were excluded from the KOrth contribution graphical analysis. For peripheral and portal blood functional analysis, STAMP was used with the Kruskal-Wallis H test, Tukey-Kramer post hoc test (0.95), and Benjamini-Hochberg FDR.

### Nuclear Magnetic Resonance (NMR)

Faecal samples from the controls and the cirrhotic patients were investigated using NMR spectroscopy to solve the spectra of complex mixtures and to recognize and quantify each component without chemical separation^82^. Briefly, NMR spectra of faecal samples were recorded at 27 °C on a Bruker AVANCE 600 spectrometer operating at a proton frequency of 600.13 MHz and equipped with a Bruker multinuclear z-gradient inverse probehead capable of producing gradients in the z-directions with a strength of 55 G/cm. The ^1^H spectra were referenced to the methyl group signals of TSP (δ = 0.00 ppm) and were acquired by co-adding 64 transients with a recycle delay of 7S. The residual HDO signal was suppressed using a presaturation. The experiment was carried out by using a 90° pulse of 11.75 μs and 32 K data points. PICRUSt spectra were transformed with 0.5 Hz line broadening and zero filling, size 65 K, manually phased, calibrated on the methyl group signals of TSP (δ = 0.00 ppm), and baseline corrected using the TOPSPIN v1.3 software. The spectra were prepared for statistical analysis by dividing the entire spectrum into small regions (0.02 ppm width), called “buckets”. Regions containing only background noise, water resonance, and the extreme regions of spectra were excluded from the buckets. The total integral (as the sum of all 418 buckets) for each spectrum was normalized to 1000. Moreover, 2D NMR experiments, namely, ^1^H-^1^H total correlation spectroscopy (TOCSY), and ^1^H-^13^C heteronuclear single quantum coherence (HSQC) were performed using the same experimental conditions previously reported^82^. The mixing time for ^1^H-^1^H TOCSY was 80 ms. The HSQC experiments were performed using a coupling constant 1JC-H of 150 Hz. A diffusion ordered spectroscopy (DOSY) experiment was performed using a bipolar LED sequence with a sine-shaped gradient of different intensities. The gradient strength was incremented in 32 steps from 2 to 95% of the maximum gradient strength (55 G/cm). The following experimental settings were used: diffusion time (Δ), 100 ms; gradient duration (δ/2), 1.1 ms, longitudinal eddy current delay, 25 ms, and gradient pulse recovery time, 100 µs. After Fourier transformation and baseline correction, the diffusion dimension was processed by means of the Bruker TOPSPIN software (version 1.3). NMR spectra were bioinformatically analysed by in-house scripts written with Python v.2.7.11, employing probabilistic quotient normalization (PQN)^83,84^, baseline removal (rolling-ball) and peak shifting (binning) correction. A matrix of normalized and corrected NMR peak areas was generated for subsequent multivariate statistical analyses.

### Data Availability

All raw data are available at the SRA database under the accession code PRJNA471972.

## Electronic supplementary material

Supplementary file

Dataset 1
