# Supplementary material for: Combining amplicon sequencing and metabolomics in cirrhotic patients highlights distinctive microbiota features involved in bacterial translocation, systemic inflammation and hepatic encephalopathy
Source: Sci Rep. 2018 May 29;8:8210. doi: 10.1038/s41598-018-26509-y (PMC5974022; doi:10.1038/s41598-018-26509-y)
Supplement: Supplementary file 1 — Supplementary file [file 41598_2018_26509_MOESM1_ESM.pdf]

SUPPLEMENTARY FILE

Combining amplicon sequencing and metabolomics in cirrhotic patients highlights distinctive microbiota features involved in bacterial translocation, systemic inflammation and hepatic encephalopathy.

Valerio Iebba[1], Francesca Guerrieri[2], Vincenza Di Gregorio[3], Massimo Levrero[2,4], Antonella Gagliardi[5], Floriana Santangelo[5], Anatoly P. Sobolev[6,7], Simone Circi[6], Valerio Giannelli[3], Luisa Mannina[6,7], Serena Schippa[5] and Manuela Merli[3*]

1. Istituto Pasteur Cenci Bolognetti Foundation, Public Health and Infectious Diseases Dept., Sapienza University of Rome, Piazzale Aldo Moro 5, 00185 Rome, Italy
2. Center for Life NanoScience@Sapienza, Istituto Italiano di Tecnologia, Rome, Italy
3. Gastroenterology, Department of Clinical Medicine, Sapienza University of Rome, Viale dell'Università 37, 00185 Rome, Italy
4. INSERM, U1052, Cancer Research Center of Lyon (CRCL), Université de Lyon (UCBL1), Centre Léon Bérard, Lyon, France
5. Public Health and Infectious Diseases Dept., Sapienza University of Rome, Piazzale Aldo Moro 5, 00185 Rome, Italy
6. Department of Drug Chemistry and Technologies, Sapienza University of Rome, Piazzale Aldo Moro 5, I-00185 Rome, Italy
7. Magnetic Resonance Laboratory "Annalaura Segre", Institute of Chemical Methodologies, CNR, via Salaria km 29.300, 00015 Monterotondo (RM) Italy

* Correspondence to:
Professor Manuela Merli, Gastroenterology, Department of Clinical Medicine, Sapienza University of Rome, Viale dell'Università 37, 00185 Rome, Italy; manuela.merli@uniroma1.it

SUPPLEMENTARY MATERIALS AND METHODS

Subjects enrolled

Forty-six patients with liver cirrhosis (aged 60.3±11.5 years, sex ratio M/F 32/13) hospitalized at the Department of Gastroenterology, University Hospital Policlinico Umberto I, were included in the study (see online supplementary table S1). The diagnosis of cirrhosis was proven through liver biopsy or based on clinical, biochemical and ultrasonographic signs. Exclusion criteria were: diagnosis of infection (based on fever, leukocytosis, elevated C Reactive protein (CRP), Erythrosedimentation Rate (ESR), procalcitonin, clinical symptoms, and positive microbiological cultures when present) , use of systemic antibiotics in the last 3 months, variceal bleeding within the last 4 weeks, alcohol or illicit drug intake within the last 3 months. Lactulose or Rifaximine therapies were not considered a cause of exclusion. Patients with any condition of immunodeficiency (HIV, immunosuppression) or with a diagnosis of hepatocellular carcinoma were excluded. Fourteen healthy age-matched individuals (aged 53.8±7.8 years, sex ratio M/F 7/6) were recruited among neighbours to serve as controls. In this group, those who were taking, or had taken in the last 3 months, medications that could potentially modify gut microbiota (antibiotics, probiotics) were excluded. Both patients and controls signed an informed consent and the protocol of the study was approved by the Hospital Ethics Committee (Protocol Number 2515/15, Rif. 3696). At the enrolment, general physical examination and vital signs were recorded. The origin of cirrhosis, previous and present complications of the disease, laboratory findings (hemogram, serum electrolyte levels, renal and liver function tests, inflammatory parameters) were collected. The severity of liver disease was evaluated by Child-Turcotte-Pugh (CTP) and model for end stage liver disease (MELD) scores. The chronic use of beta-blockers, lactulose, proton pump inhibitors (PPI) and other drugs possible influencing gut microbiota was recorded. Patients included in the study and healthy controls gave a fresh stool sample that was promptly stored at -80°C. Cirrhotic patients also underwent serum collection of peripheral vein blood samples (2 mL) for cytokines titration (TNF-α, IL1β, IL6) and blood microbiota assessment. Portal blood (2 mL) was taken from seven cirrhotic patients admitted for TIPS procedure, and underwent the same analyses of peripheral blood. Both peripheral and portal blood samples were immediately frozen upon collection at -80°C. In patients and controls undergoing a colonoscopy for prevention of colorectal carcinoma as indicated by clinical guidelines, or due to a general work up, before being admitted to liver transplant list, a mucosal biopsy (cecum) was obtained for the assessment of intestinal microbiota adherent to the mucosa, and immediately stored at -80°C. All retrieved demographic and clinical parameters were anonymously used to build a matrix employed for subsequent multivariate statistical analysis.

ELISA tests

Evaluation of peripheral and portal blood levels of IL-6, TNF-α, and IL1-β cytokines was carried out by Enzyme-Linked ImmunoSorbent Assay (ELISA). Briefly, 2 ml of peripheral and portal blood were collected as specified above in a test tube with anticoagulant, and centrifuged at 3000 rpm for 10 minutes. One-hundred μL of supernatant were used to evaluate cytokines level by enzyme immunoassays carried out with commercial kits (Human ELISA Ready-SET-Go!, cat# 88-7066-22 for IL-6, cat# 88-7346-22 for TNF-α, cat# 88-7261-22 for IL1-β, eBioscience, San Diego, CA, United States), and assays were performed in triplicate following the manufacturer's instructions. Plates (96-well ELISA plate, Corning Costar 9018, included in the kit) were read at 450 nm (subtracted by 570 nm readings) and intensity measurements were analyzed with an online software (http://www.elisaanalysis.com/) to retrieve values expressed as pg/mL (a four-parameters logistic curve was used for standard curve interpolation). A matrix of ELISA data was

generated for subsequent multivariate statistical analysis. Mann-Whitney U test was used to assess significant comparisons ($P \leq 0.05$).

Microbiota characterization of biopsies, blood and stool samples

Biopsies underwent a first wash (30s mid-speed vortex) with 0.016% dithioerythritol (DTT, cat#D0632, Sigma-Aldrich, Milan, Italy) in phosphate-buffered saline (PBS, cat#AU-L0615-500, Aurogene, Rome, Italy) to remove the mucus, then washed three more times in PBS. Total DNA from stool samples (200 mg / each), from biopsies (15 mg /each) and from peripheral/portal blood (200 µl / each) was automatically extracted with Maxwell® RSC Instrument (Promega. Wisconsin, USA, kit #AS1400). For all three kind of samples, manufacturer's protocol was modified by incubating them with proteinase K at 56 °C, followed by a 4 hours incubation at 37 °C with 2 mg/ml (final concentration) of lysozyme (cat# L6876, Sigma-Aldrich, Milan, Italy) to ensure a proper disruption of Gram-positive bacterial species. Next Generation Sequencing (NGS) of 16S rDNA V3-V4 regions amplicons was thus carried out on a total of 109 samples so divided: i) cecum samples (17 HC, 6 controls); ii) stool samples (35 HC, 14 controls); iii) blood samples (30 HC peripheral, 7 HC portal). Samples underwent robotic library preparation and sequencing as per Illumina 16S metagenomics standardized operational workflow (16S Metagenomic Sequencing Library Preparation, Part # 15044223 Rev. B). Each 16s library was checked for size with Agilent 2200 Tapestation (Agilent Technologies, Santa Clara, CA, United States) and quantified with Qubit 2.0 fluorometer using Qubit dsDNA HS Assay Kit (cat# Q32851, Thermo Fisher Scientific, MA, United States). Sequencing was performed at Italian Institute of Technology (https://www.iit.it/it/centers/clns-sapienza) with Illumina MiSeq platform, Reagent Kit v3 (cat# MS-102-3003, Illumina, San Diego, CA, United States), 2x300 paired-end, 600-cycle. Raw FASTQ files were analyzed with Mothur pipeline v.1.38.1 [1] for quality check and filtering (sequencing errors, chimerae) on a Workstation CELSIUS R940 (Fujitsu, Minato-ku Tokyo, Japan). Filtered reads (9209053 in total, on average 84487 per sample, see online supplementary table S3) were clustered into Operational Taxonomical Units (OTUs), after singletons/doubletons elimination, by de novo OTU picking at 97% pair-wise identity using standardized parameters and SILVA rDNA Database v.1.19 [2] for alignment. In all, 1990 OTUs were identified. Given the high heterogeneity of the six datasets (biop_cirr, biop_ctrl, feces_cirr, feces_ctrl, periph_blood, portal_blood) in terms of OTUs and filtered-quality reads numbers, all samples were normalized (rarefied) to the number of reads present in the less rich one (3101 reads for a portal blood sample). Sample coverage was computed with Mothur and resulted to be on average equal to 99% for all samples (mean±SDM, 99.1%±0.5%), thus meaning a suitable normalization procedure for subsequent analyses. Analysis Of Molecular Variance [3] (AMOVA, which represents the difference of datasets' centroids), Homogeneity Of Molecular Variance (HOMOVA, representing the difference of datasets' standard deviations), parsimony test, LEfSe [4], and Random Forest (RF) error rate were computed with Mothur v.1.38.1.

OTUs species assignment and multivariate statistical analyses

Bioinformatic and statistical analyses on recognized OTUs were performed with Python v.2.7.11. The most representative and abundant read within each OTU (as evidenced in the previous step with Mothur v.1.38.1) underwent a nucleotide Blast using the National Center for Biotechnology Information (NCBI) Blast software (ncbi-blast-2.3.0) and the latest NCBI 16S Microbial Database (ftp://ftp.ncbi.nlm.nih.gov/blast/db/). Retrieved species (first 500

OTUs) had the following Blast parameters values (mean±SDM) (see online supplementary file): E-value ($1.82*10^{-85}$±$3.59*10^{-85}$), total score (703.5±103.6), percentage identity (94.9±4.0) and mismatches (21.2±16.3). A matrix of bacterial relative abundances was built at each taxon level (phylum, class, order, family, genus, species, OTUs) for subsequent multivariate statistical analyses. Measurements of α diversity (within sample diversity) such as observed_otus and Shannon index, were calculated at OTUs level using the SciKit-learn package v.0.4.1. Exploratory analysis of β-diversity (between sample diversity) was calculated using the Yue & Clayton measure of dissimilarity (θ) calculated with Mothur and represented in Principal Coordinate Analyses (PCoA), while for Hierarchical Clusterization Analysis (HCA) 'Bray-Curtis' metrics and 'complete linkage' method were used implementing in-house scripts (Python v.2.7.11). In order to compare the microbiota taxa (at mean relative abundance level ≥ 0.5%) with demographics/clinical, NMR metabolomics, and ELISA datasets, a multivariate statistical Pearson correlation analysis (and related *P* values) was performed with in-house Python scripts. Pearson correlation matrices (metric=Bray-Curtis, method=complete linkage) were generated also for intra- and inter-dataset (biop_cirr, biop_ctrl, feces_cirr, feces_ctrl, periph_blood, portal_blood, NMR metabolomics) cluster generation and positive/negative correlation coefficient discovery. Mann-Whitney U and Kruskall-Wallis tests were employed to assess significance for pair-wise or multiple comparisons, respectively, taking into account a P value less than or equal to 0.05 as significant. Cross-correlation Pearson matrices for network analysis (metric=Bray-Curtis, method=complete linkage) were generated with in-house scripts (Python v.2.7.11) and visualized with Gephi v.0.9.1 [5], taking into account OTUs having a mean relative abundance ≥ 0.5% and Pearson correlation coefficients $-0.7 > r > 0.7$ as previously reported [6], after Benjamini-Hochberg FDR at 10%. A network analysis was performed on each dataset (biop_cirr, biop_ctrl, feces_cirr, feces_ctrl, periph_cirr, portal_cirr, NMR metabolomics, and merged 16S/NMR) using co-occurrences and visual representation as proposed by current guidelines [7–11]. Degree value, measuring the in/out number of edges linked to a node, and betweenness centrality, measuring how often a node appears on shortest paths between pairs of nodes in a network, were computed with Gephi v.0.9.1. Networks' density and modularity were also computed with Gephi v.0.9.1: density would reflect the 'shrinkage' of network itself, while modularity the capability of network in hosting different (and various) communities within itself. Intra-network communities (even for Functional Metagenomics Communities, or FMCs) were retrieved using the Blondel community detection algorithm [12], by means of randomized composition and edge weights, and a resolution equal to 1 [13]. Clustering validation (K-means++ [14], Birch [15], Affinity Propagation [16]) and performance measures (Silhouette score [17], Calinski-Harabasz score [18]) were employed to confirm intra-network clusterization into communities. To find correlations among bacterial species, genes (KOs, effect size ≥ 0.47), fecal metabolites and presence/absence of hepatic encephalopathy (HE), we used logistic regression ($-\infty < \beta < \infty$), randomized lasso ($0 < \beta < 1$), elastic net ($-\infty < \beta < \infty$), and SGD classifier ($-\infty < \beta < \infty$) within Python SciKit learn module [19,20] on mean-centered and unit-variance dataframes. Odds ratios (OR) were computed from logistic regression coefficients (β) with the formula $OR=2^{\beta}$ [21].

Metagenome prediction and pathway analysis

Biom file was generated with Mothur v.1.38.1 using Greengenes database (v. 13_5_99), and used with PICRUSt 1.0.0 (Phylogenetic Investigation of Communities by Reconstruction of Unobserved States) [22] with default parameters, in order to predict Kyoto Encyclopedia of Genes and Genomes (KEGG) orthologs (KOs) from 16S V3-V4 amplicon data. Specific KOs related to significant NMR metabolites were bioinformatically assigned to each sample by

means of KEGG online website (http://www.genome.jp/kegg/ko.html) and Integrated Database Retrieval System (http://www.genome.jp/dbget/), taking into account Orthology and Reactions databases for a refined search. STAMP [23] was then utilized employing two-sided Welch's t-test and η^2 (effect size) to detect specific KOs having discriminant power ($P \leq 0.05$) and functional relationship to NMR data of cirrhotic/controls fecal samples. Mean relative KO abundances, normalized per sample number, were computed with in-house Python scripts, and those lower than a definite threshold ($2*10^{-6}$ % for phylum, $5*10^{-6}$ % for genus) were excluded from KO contribution graphical analysis. For peripheral and blood functional analysis, STAMP was used with Kruskal-Wallis H test, Tukey-Kramer post-hoc test (0.95), and 10% Benjamini-Hochberg FDR.

Nuclear Magnetic Resonance (NMR)

Fecal samples of controls and cirrhotics were investigated using NMR spectroscopy to solve spectra of complex mixtures and to recognize and quantify each component without chemical separation [24]. Briefly, NMR spectra of fecal samples were recorded at 27°C on a Bruker AVANCE 600 spectrometer operating at the proton frequency of 600.13 MHz and equipped with a Bruker multinuclear z-gradient inverse probehead capable of producing gradients in the z-directions with a strength of 55 G/cm. 1H spectra were referenced to methyl group signals of TSP (δ=0.00 ppm) and they were acquired by co-adding 64 transients with a recycle delay of 7 s. The residual HDO signal was suppressed using a pre-saturation. The experiment was carried out by using a 90° pulse of 11.75 µs, and 32K data points. 1H spectra were transformed with 0.5 Hz line broadening and zero filling, size 65K, manually phased, calibrated on methyl group signals of TSP (δ=0.00 ppm), and baseline corrected using the TOPSPIN v1.3 software. Spectra were prepared for statistical analysis by dividing the entire spectrum into small regions (0.02 ppm width), called "buckets". Regions with only background noise, the water resonance, and the extreme regions of spectra were excluded from the buckets. The total integral (as the sum of all 418 buckets) for each spectrum was normalized to 1000. 2D NMR experiments, namely, 1H-1H TOtal Correlation SpectroscopY (TOCSY), and 1H-13C Heteronuclear Single Quantum Coherence (HSQC) were performed using the same experimental conditions previously reported [24]. The mixing time for 1H-1H TOCSY was 80 ms. HSQC experiments were performed using a coupling constant 1JC-H of 150 Hz. Diffusion Ordered SpectroscopY (DOSY) experiment was performed using bipolar LED sequence with sine-shaped gradient of different intensities. The gradient strength was incremented in 32 steps from 2 to 95% of the maximum gradient strength (55 G/cm). The following experimental settings were used: diffusion time, Δ was 100 ms, gradient duration, $\delta/2$ was 1.1 ms, the longitudinal eddy current delay was 25 ms, the gradient pulse recovery time was set to 100 µs. After Fourier transformation and baseline correction, the diffusion dimension was processed by means of the Bruker TOPSPIN software (version 1.3). NMR spectra were bioinformatically analyzed by in-house scripts made with Python v.2.7.11, employing probabilistic quotient normalization (PQN) [25,26], baseline removal (rolling-ball) and peak shifting (binning) correction. A matrix of normalized and corrected NMR peaks' areas was generated for subsequent multivariate statistical analyses

Supplementary table S1. Demographic and clinical characteristics of patients with liver cirrhosis.

Number of Pts.	46
Age (years)	60.3 ± 11.5
Sex (M/F)	32/13
Aetiology of cirrhosis (virus/alcohol/virus+alcohol/other), n° (%)	14 (31)/13 (28.9)/ 5 (11.1)/13 (28.9)
CTP Class (A/B/C) n°(%)	14 (32.5)/23 (53.5)/6 (14)
MELD score	13.5 ± 4
Presence of oesophageal varices, n° (%)	38 (86.3%)
Ascites, n° (%)	24 (54.5%)
Hepatorenal Syndrome, n° (%)	2 (4.5%)
Hepatic Encephalopathy, n° (%)	8 (18.2%)
Albumin (g/dl)	3.1 ± 0.5
Sodium (mEq/L)	136 ± 4
Bilirubin (mg/dl)	1.7 ± 1
Creatinine (mg/dl)	1 ± 0.4
INR	1.4 ± 0.3
VES (mm/h)	29 ±2
CRP (mg/dl)	1.6 ± 3
Death during the recovery, n° (%)	5 (11.3)
Hepatocellular Carcinoma, n° (%)	16 (36)
Median Arterial Pressure (mmHg)	83±11
Cardiac frequency (bpm)	72 ± 13
White Blood Cells (n°/mm3)	3987 ± 2237
GPT (UI/ml)	38.6±33
PLT (n/mm3)	94098±10049
Hemoglobin (g/dl)	10.6±2
B-blockers, n° (%)	34 (74)
Diuretic therapy, n° (%)	27 (61)
Proton Pump Inhibitors, n° (%)	33 (74)
Lactulose, n°(%)	17 (38)
Rifaximine, n° (%)	2 (4.5)

Supplementary table S2. Reads and OTUs obtained from MiSeq runs for each dataset.

	biop_cirr	biop_ctrl	faeces_cirr	faeces_ctrl	periph_cirr	portal_cirr	Total (or avg/sample)
samples (n)	17	6	35	14	30	7	109
raw_reads	2270128	1254162	7861085	6444647	3575803	767706	22173531
raw_reads/sample	133537	209027	224602	460332	119193	109672	203427/sample
FQ_reads[a]	936810	510400	3477986	2360223	1598877	324757	9209053
FQ_reads/sample	55106	85067	99371	168587	53296	46394	84487/sample
OTUs[b]	448	297	353	256	432	204	1990
OTUs/sample	26	49	10	18	14	29	146

a) FQ, filter-quality with Mothur pipeline v.1.38.1
b) OTUs, Operational Taxonomical Units

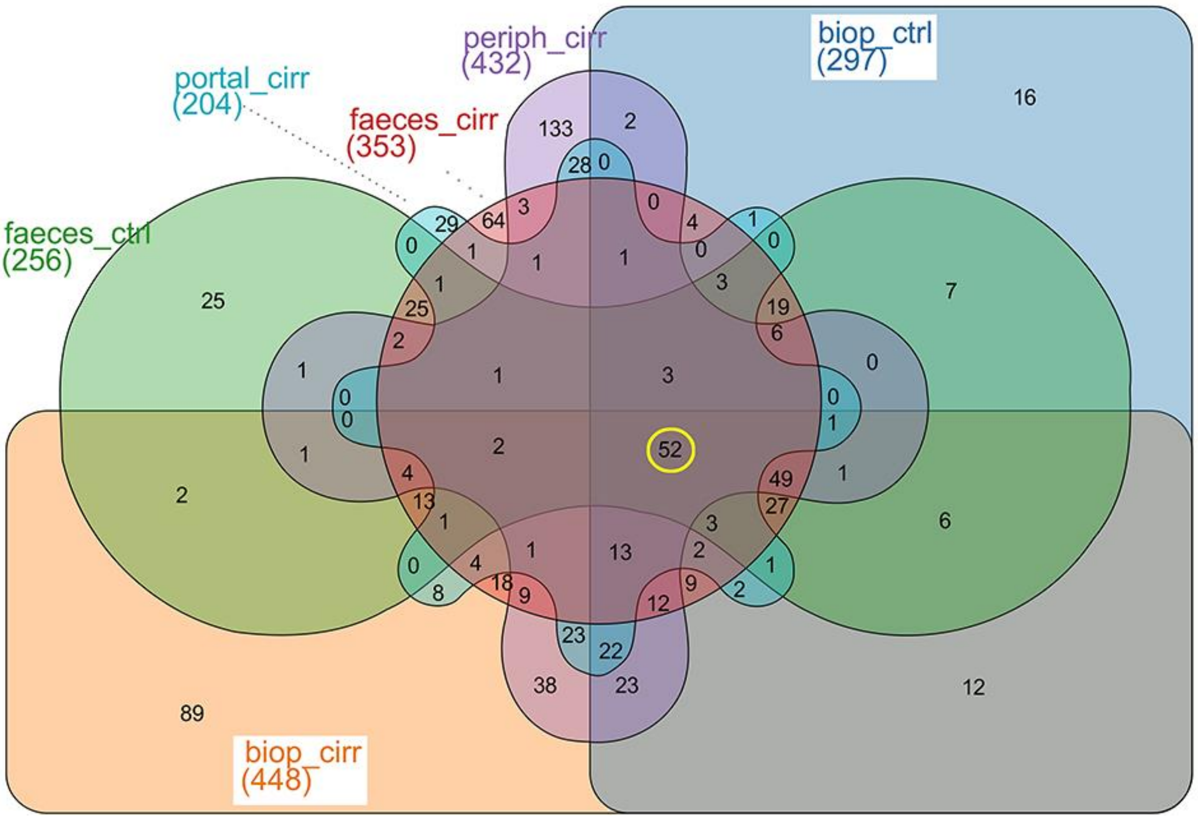

Supplementary figure S1. Distribution of OTUs. Venn diagram reports distribution of all OTUs (enclosed numbers) among the six datasets: biop_cirr, biop_ctrl, feces_cirr, feces_ctrl, periph_cirr, portal_cirr (panel A). The ‘core’ microbiota for all datasets is composed by 52 OTUs (yellow circle).

Supplementary table S3. Mean relative abundances (≥ 0.5%) of bacterial Phyla. In bold are significant comparisons (*P*≤0.05).

Phylum (relative abundance %)	Biopsies cirr	Biopsies ctrl	*P* value (MW) biopsies	Feces cirr	Feces ctrl	*P* value (MW) faeces	Peripheral blood	Portal blood	*P* value (MW) blood
Actinobacteria	2.88	1.04	0.278	1.64	0.52	0.357	3.62	4.69	0.698
Bacteroidetes	30.19	38.96	0.310	42.78	51.71	0.432	9.12	17.43	0.393
Firmicutes	34.42	41.30	0.100	34.87	39.39	0.104	5.43	11.08	0.404
Fusobacteria	1.21	3.20	0.273	0.63	0.00	**0.005**	0.04	0.00	0.110
Proteobacteria	28.26	11.34	0.074	12.10	6.42	0.199	81.19	65.11	0.427
Verrucomicrobia	2.26	3.57	0.401	7.18	1.41	**0.013**	0.36	0.97	0.397
unclassified	0.54	0.44	0.916	0.48	0.37	0.127	0.09	0.38	**0.043**

MW = Mann-Whitney U test

Supplementary table S4. Mean relative abundances of representative bacterial species described in the manuscript. In bold are significant *P*≤0.05. MW = Mann-Whitney U test

Species	Biop Cirr	Biop Ctrls	*P* value (MW) biopsies	Feces Cirr	Feces Ctrl	*P* value (MW) faeces	Peripheral blood	Portal blood	*P* value (MW) blood
Acinetobacter guillouiae	0.011	0.011	0.527	0.002	0.000	0.383	25.909	14.364	0.561
Akkermansia muciniphila	2.261	3.569	0.401	7.179	1.387	**0.015**	0.356	0.967	0.397
Alistipes onderdonkii	0.006	0.102	**0.017**	0.603	0.723	**0.014**	0.003	0.000	0.679
Bacteroides coprocola	0.044	0.000	0.621	1.284	0.689	0.844	0.057	0.765	**0.001**
Bacteroides coprophilus	0.269	0.193	**0.042**	1.284	0.297	0.264	0.084	0.143	0.922
Bacteroides plebeius	0.021	1.403	0.720	0.322	2.741	0.112	0.226	0.138	0.980
Bacteroides stercoris	0.085	0.161	0.410	1.182	0.403	0.332	0.016	0.000	0.520
Bacteroides uniformis	0.051	1.129	**0.021**	2.066	5.609	**0.005**	0.024	0.332	0.151
Bacteroides vulgatus	3.908	2.601	0.806	16.977	8.566	**0.048**	0.285	1.216	0.218
Barnesiella intestinihominis	7.910	8.685	0.834	0.309	1.963	**1.46E-07**	0.964	1.907	0.497

Barnesiella viscericola	8.341	8.444	0.834	0.006	0.000	0.146	1.521	2.916	0.426
Bifidobacterium breve	0.577	0.000	0.621	0.270	0.000	**0.040**	0.000	0.000	1
Brevibacillus brevis	4.921	1.123	0.599	0.000	0.000	1	0.055	0.000	0.215
Campylobacter concisus	0.027	0.005	0.495	0.156	0.000	0.078	0.000	0.115	**0.045**
Clostridium clostridioforme	0.121	0.097	0.858	0.427	0.044	**0.016**	0.001	0.000	0.679
Clostridium xylanolyticum	0.091	0.537	0.087	0.081	4.457	**1.66E-06**	0.272	1.037	0.112
Elizabethkingia miricola	0.956	0.124	**0.005**	0.002	0.000	0.557	2.312	3.068	0.669
Enterobacter cloacae	1.167	0.124	**0.010**	1.559	0.000	**0.003**	5.475	3.695	0.394
Escherichia fergusonii	7.690	3.628	0.195	6.627	0.039	**0.003**	0.377	1.281	**0.047**
Faecalibacterium prausnitzii	0.317	4.294	**0.003**	1.756	5.471	**0.005**	0.200	0.378	0.094
Gemmiger formicilis	0.028	0.537	**0.002**	0.818	2.230	**0.008**	0.169	0.088	0.478
Oscillibacter ruminantium	0.347	0.951	**0.019**	0.717	2.948	**2.60E-05**	0.177	0.783	**0.019**
Propionibacterium acnes	1.444	0.306	**0.019**	0.002	0.000	0.383	2.856	3.515	0.641
Pseudomonas fluorescens	1.969	0.382	**0.012**	0.002	0.002	0.873	31.966	20.731	0.342
Roseburia faecis	0.163	1.580	0.107	0.814	1.734	**0.008**	0.049	0.000	0.173
Ruminococcus gnavus	2.969	2.268	0.362	0.648	0.007	**0.001**	0.199	0.217	0.686
Ruminococcus torques	0.757	1.811	**0.049**	0.401	0.230	0.458	0.011	0.000	0.520
Sphingomonas paucimobilis	10.602	2.370	0.441	0.001	0.005	0.143	8.177	14.581	0.185
Veillonella dispar	0.943	0.204	0.342	9.684	0.101	**8.68E-05**	0.180	0.723	0.118

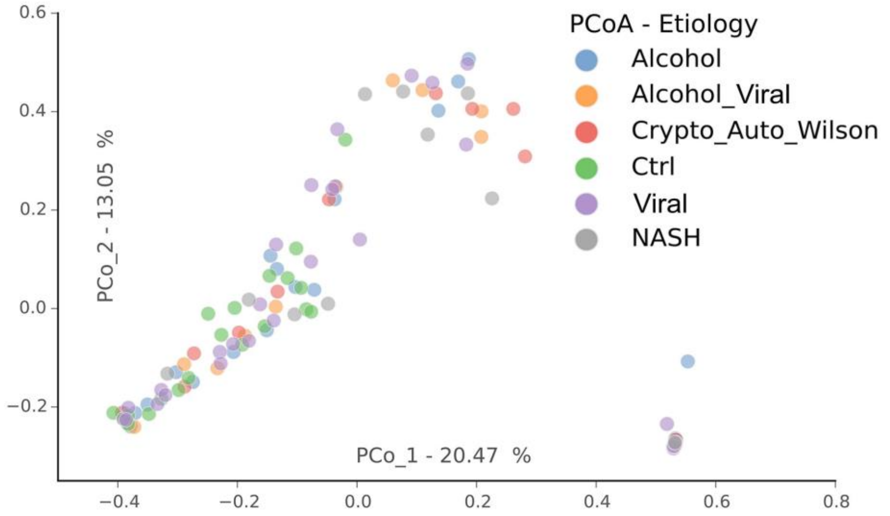

Supplementary figure S2. Beta-diversity of all samples divided by etiology. PCoA analysis was performed on a distance matrix based on Yue & Clayton measure of dissimilarity, dividing all samples (n=109) by etiology. PCoA classified patients by etiology (Amova P=0.003, Bonferroni pair-wise error rate: 0.0033, RF error rate: 0.577), even if not so indicative due to the heterogeneous origin of samples. PCoA evidenced significant differences for the following comparisons: Alcohol-Ctrl (P<0.001), Alcohol_Viral-Ctrl (P=0.003), Crypto_Auto_Wilson-Ctrl (P=0.001), Viral-Ctrl (P<0.001) and NASH-Ctrl (P<0.001).

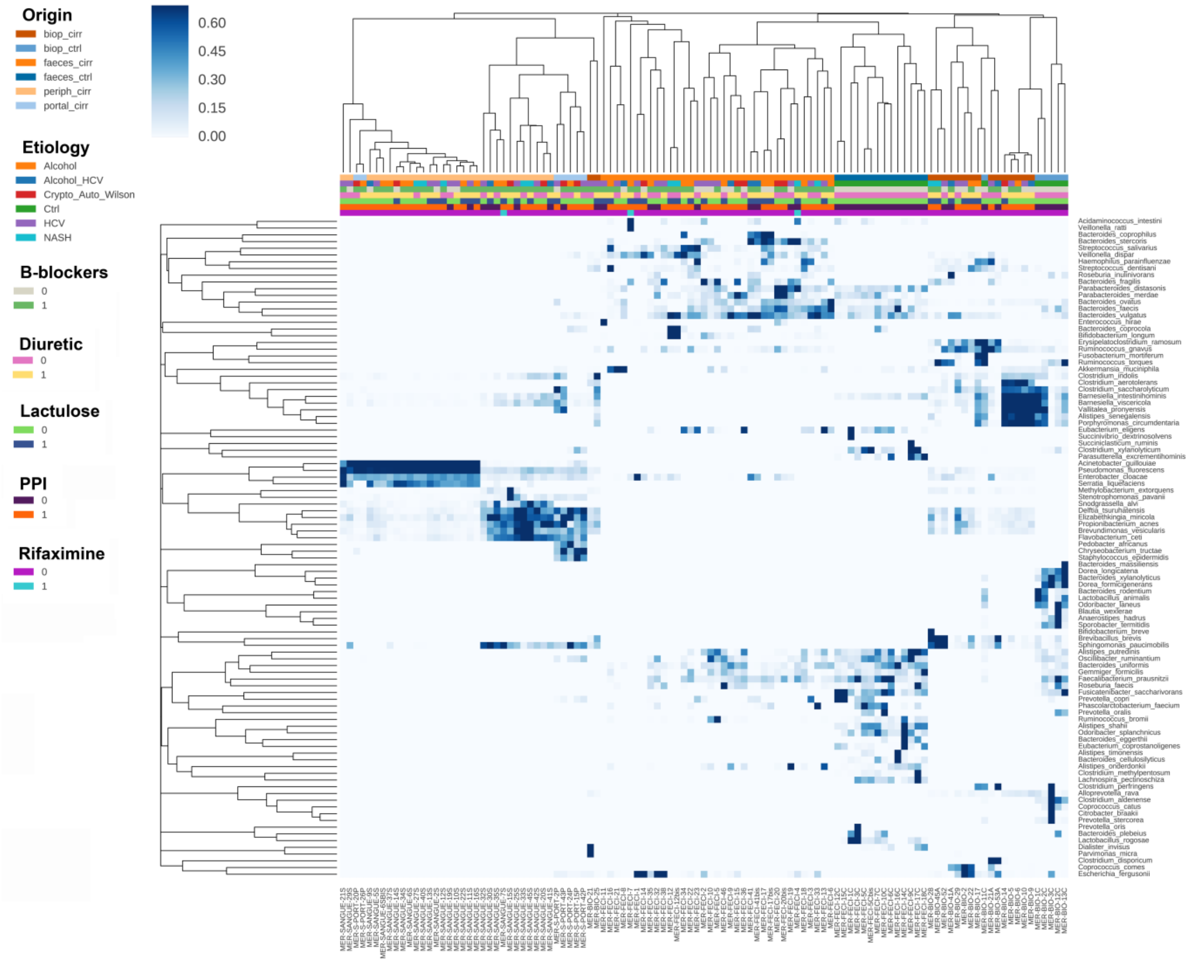

Supplementary figure S3. Hierarchical Clusterization Algorithm (HCA) of cirrhotic and control samples. HCA was performed on all samples (x axis) taking into account the bacterial species having a mean relative abundance equal to or higher than 0.5% (y axis). Both dendrograms were generated with Bray-Curtis similarity and complete-linkage agglomeration. Colors on x axis (samples) refer to the different samples' origin, cirrhosis etiology and drug treatments (beta-blockers, diuretic, lactulose, PPI, rifaximine).

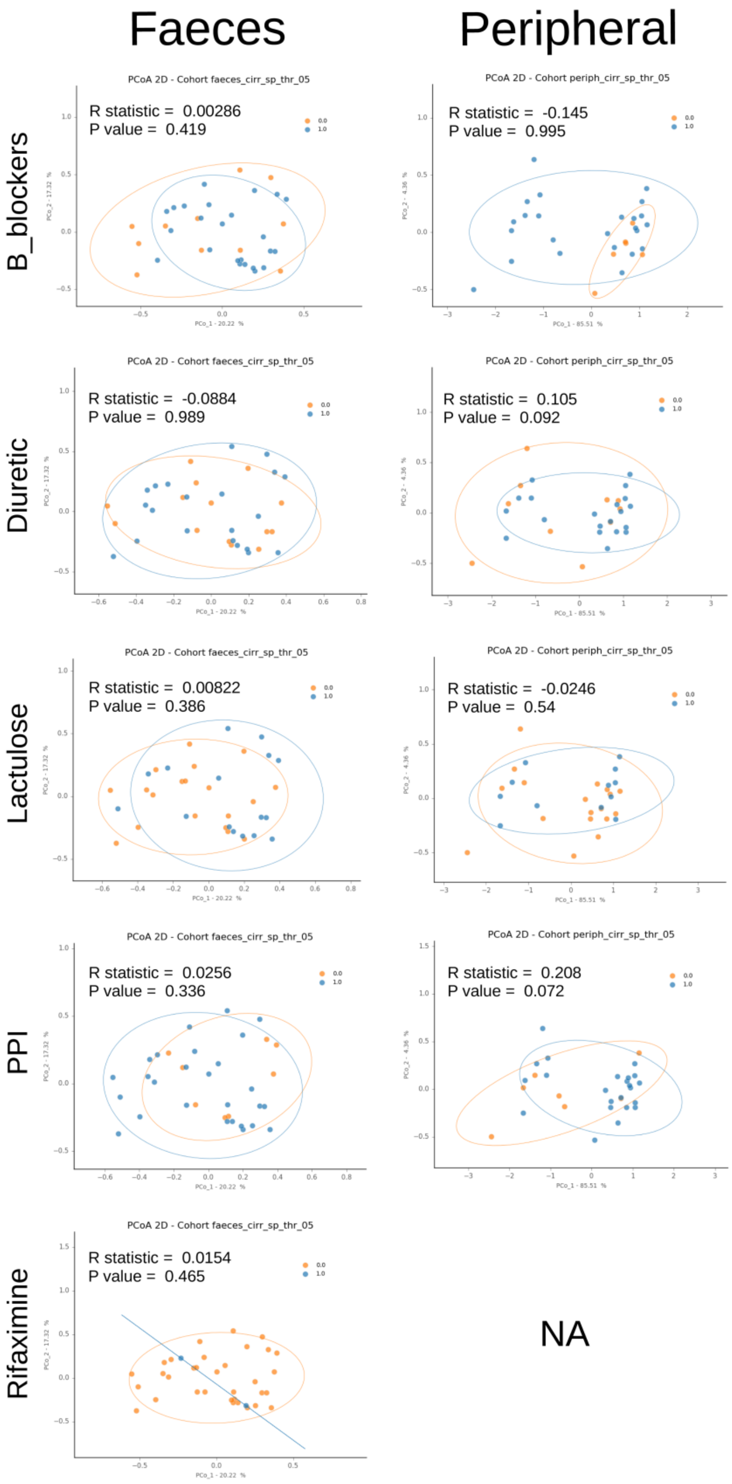

Supplementary figure S4. Beta-diversity of all samples divided by drug treatment. PCoA analysis was performed on a distance matrix based on Bray-Curtis measure of dissimilarity, dividing fecal and peripheral blood cirrhotic samples by five drug treatments (beta-blockers, diuretic, lactulose, PPI, rifaximine). R statistic and P values were retrieved from Anosim analysis.

Supplementary table S5. Significant KEGG orthologues with positive effect size (higher proportion) in cirrhotics

KEGG ortholog[a)]	Metabolic pathways	Gene name	Description	Difference in mean % proportion (cirrhotic vs ctrls)	*P* value
**K02770**	ko02060 Phosphotransferase system (PTS)	PTS-Fru-EIIC/fruA	PTS system, fructose-specific IIC component	0.005	0.042
**K02822**	ko02060 Phosphotransferase system (PTS)	PTS-Ula-EIIB/ulaB/sgaB	PTS system, ascorbate-specific IIB component [EC:2.7.1.194]	0.004	0.044
**K01447**	ko01000 Enzymes	cwlA/xlyA/xlyB	N-acetylmuramoyl-L-alanine amidase [EC:3.5.1.28]	0.004	0.032
**K00852**	ko00030 Pentose phosphate pathway	rbsK	ribokinase [EC:2.7.1.15]	0.004	0.048
K06193	ko01120 Microbial metabolism in diverse environments	phnA	protein PhnA	0.003	0.040
K07341	ko02048 Prokaryotic defense system	doc	death on curing protein	0.002	0.042
K10563	ko03410 Base excision repair	mutM/fpg	formamidopyrimidine-DNA glycosylase [EC:3.2.2.23 4.2.99.18]	0.002	0.034
K00940	ko00230 Purine metabolism ko00240 Pyrimidine metabolism	ndk/NME	nucleoside-diphosphate kinase [EC:2.7.4.6]	0.002	0.037
K04564	ko04211 Longevity regulating pathway	SOD2	superoxide dismutase, Fe-Mn family [EC:1.15.1.1]	0.001	0.028
K03637	ko00790 folate biosynthesis	moaC/CNX3	cyclic pyranopterin monophosphate synthase [EC:4.6.1.17]	0.001	0.039

a) In bold are the orthologues within the first 20 KOs.

Supplementary table S6. Significant KEGG orthologues related to NMR results, all with negative effect size (lower proportion) in cirrhotics

KEGG ortholog[a)]	Metabolic pathways	Gene name	Description	Difference in mean % proportion (cirrhotic vs ctrls)	*P* value
K00043	ko00650 Butanoate<br>ko01200<br>Carbon metabolism	gbd	4-hydroxybutyrate dehydrogenase [EC:1.1.1.61]	< -0.001	0.033
K00109	ko00650 Butanoate	E1.1.99.2	2-hydroxyglutarate dehydrogenase [EC:1.1.99.2]	< -0.001	0.043
K01028	ko00650 Butanoate	scoA	3-oxoacid CoA-transferase subunit A [EC:2.8.3.5]	-0.001	0.039
K01666	ko00360<br>Phenylalanine metabolism	mhpE	4-hydroxy 2-oxovalerate aldolase [EC:4.1.3.39]	-0.001	0.041
K02688	ko03000<br>Transcription;<br>Transcription factors	prpR	transcriptional regulator, propionate catabolism operon regulatory protein	< -0.001	0.026
K03416	ko00640 Propanoate metabolism	E2.1.3.1-5S	methylmalonyl-CoA carboxyltransferase 5S subunit [EC:2.1.3.1]	< -0.001	0.043
K15036	ko00640 Propanoate metabolism<br>ko01200<br>Carbon metabolism	K15036	acetyl-CoA/propionyl-CoA carboxylase [EC:6.4.1.36.4.1.2]	< -0.001	0.035
K01844	ko00310<br>Lysine degradation	kamD	beta-lysine 5,6-aminomutase alpha subunit [EC:5.4.3.3]	< -0.001	0.044
K00306	ko00310<br>Lysine degradation	PIPOX	sarcosine oxidase / L-pipecolate oxidase [EC:1.5.3.71.5.3.1]	< -0.001	0.043
K00831	ko00680<br>Methane metabolism<br>ko00260<br>Glycine, serine and threonine metabolism<br>ko01200<br>Carbon metabolism	serC/PSAT1	phosphoserine aminotransferase [EC:2.6.1.52]	-0.002	0.031
**K02203**	ko00680<br>Methane metabolism<br>ko00260<br>Glycine, serine and threonine metabolism	thrH	phosphoserine / homoserine phosphotransferase [EC:3.1.3.3 2.7.1.39]	-0.004	0.046
K03390	ko00680<br>Methane metabolism<br>ko01200<br>Carbon metabolism	hdrC	heterodisulfide reductase subunit C [EC:1.8.98.1]	< -0.001	0.046
K06034-K13039	ko00680<br>Methane metabolism	comD-comE	sulfopyruvate decarboxylase subunit alpha and beta [EC:4.1.1.79]	< -0.001	0.043
**KEGG ortholog[a)]**	**Metabolic pathways**	**Gene name**	**Description**	**Difference in mean % proportion (cirrhotic vs ctrls)**	***P* value**
K11780-	ko00680	cofG-cofH	FO synthase subunit 1 and 2	< -0.001	0.043

K11781	Methane metabolism		[EC:2.5.1.77]		
K14940	ko00680 Methane metabolism	cofF	gamma-F420-2:alpha-L-glutamate ligase [EC:6.3.2.32]	< -0.001	0.030
K14028-K14029	ko00680 Methane metabolism ko00010 Glycolysis / Gluconeogenesis ko01200 Carbon metabolism	mdh1/mxaF - mdh2/mxaI	methanol dehydrogenase (cytochrome c) subunit 1 and 2 [EC:1.1.2.7]	< -0.001	0.045
K14081	ko00680 Methane metabolism ko01200 Carbon metabolism	mtaC	methanol corrinoid protein	< -0.001	0.024
K00138	ko00010 Glycolysis / Gluconeogenesis	aldB	aldehyde dehydrogenase [EC:1.2.1.-]	< -0.001	0.039
K06859	ko00010 Glycolysis / Gluconeogenesis ko01200 Carbon metabolism	pgi1	glucose-6-phosphate isomerase, archaeal [EC:5.3.1.9]	< -0.001	0.043
K00029	ko01200 Carbon metabolism	maeB	malate dehydrogenase (oxaloacetate-decarboxylating)(NADP+) [EC:1.1.1.40]	-0.002	0.045
K00031	ko01200 Carbon metabolism	IDH1/IDH/ icd	isocitrate dehydrogenase [EC:1.1.1.42]	-0.003	0.048
K00051	ko01200 Carbon metabolism	E1.1.1.82	malate dehydrogenase (NADP+) [EC:1.1.1.82]	< -0.001	0.041
K00282-K00283	ko01200 Carbon metabolism	gcvPA - gcvPB	glycine dehydrogenase subunit 1 and 2 [EC:1.4.4.2]	-0.001	0.039
K14451	ko01200 Carbon metabolism	mcl2	(3S)-malyl-CoA thioesterase [EC:3.1.2.30]	< -0.001	0.047
K14472	ko01200 Carbon metabolism	smtB	succinyl-CoA:(S)-malate CoA-transferase subunit B [EC:2.8.3.22]	< -0.001	0.041
K15016	ko01200 Carbon metabolism	K15016	enoyl-CoA hydratase / 3-hydroxyacyl-CoA dehydrogenase [EC:4.2.1.17 1.1.1.35]	< -0.001	0.043
K15019	ko01200 Carbon metabolism	K15019	3-hydroxypropionyl-coenzyme A dehydratase [EC:4.2.1.116]	< -0.001	0.041

a) In bold are the orthologues within the first 20 KOs.

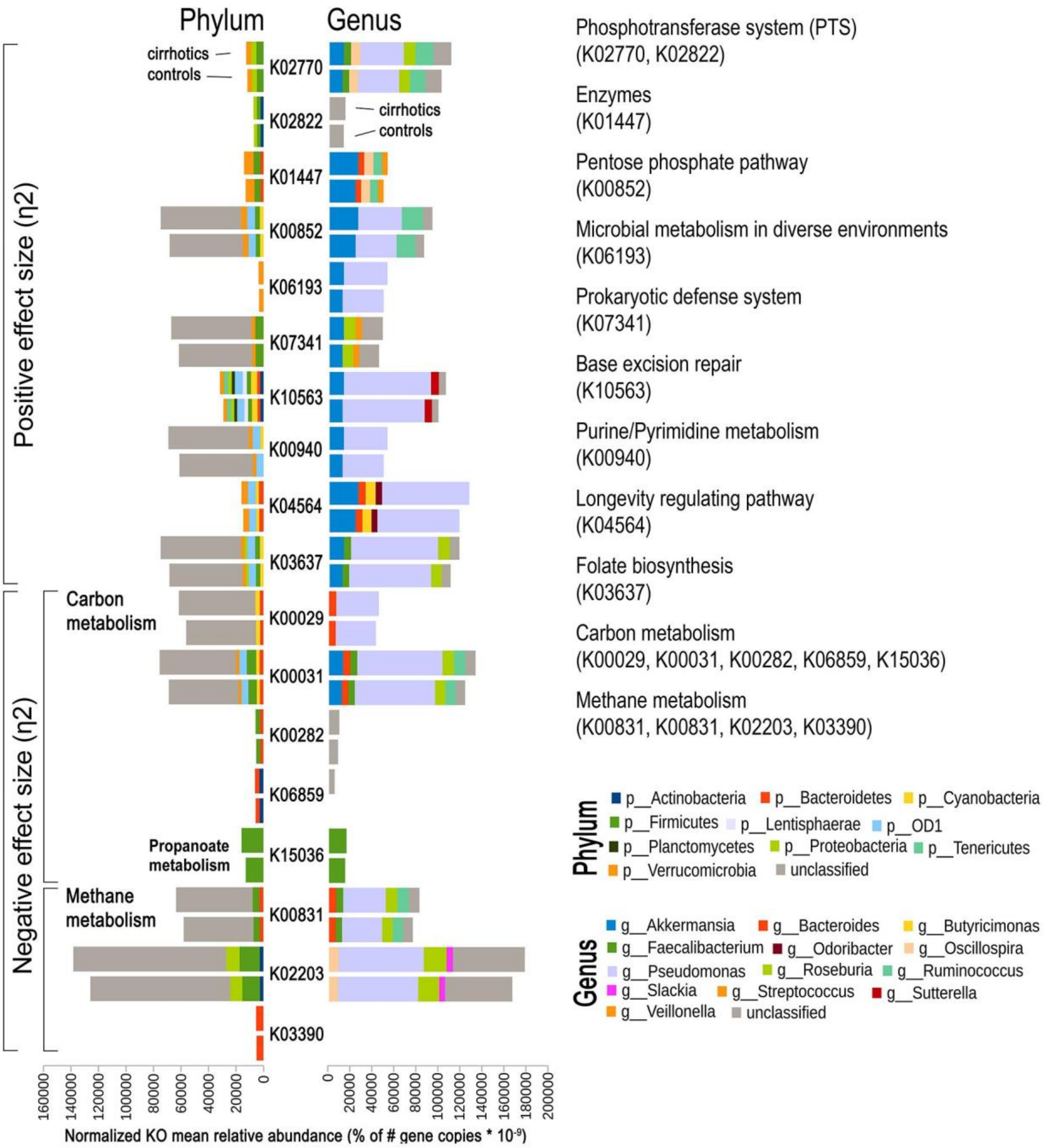

Supplementary figure S5. Picrust analysis for phyla and genera contributions. Picrust analysis (panel C) was used to compute the average relative contributions of phyla and genera for selected KOs. The 10 KOs with higher percentage contribution in cirrhotics (positive effect size - overrepresented, see online supplementary table S4) were reported in descendent order of effect size, while 8 KOs with lower percentage contribution in cirrhotics (negative effect size - underrepresented, see online supplementary table S5), involved in carbon (pathway ko01200) and methane (pathway ko00680) metabolism, were reported in numerical ascending order.

Supplementary table S7. Clustering validation and performance measures of controls and cirrhotics FMNs into FMCs

	Control feces (16S + NMR)				Cirrhotics feces (16S + NMR)			
AP	5				2			
#_clusters	Silhouette_score (K-means)	Silhouette_score (Birch)	Calinski_score (K-means)	Calinski_score (Birch)	Silhouette_score (K-means)	Silhouette_score (Birch)	Calinski_score (K-means)	Calinski_score (Birch)
2	0.257	0.255	22.16	20.05	**0.191**	**0.187**	**17.26**	**16.82**
3	0.331	0.320	25.33	23.74	0.155	0.163	12.91	12.85
4	0.408	0.408	32.09	32.56	0.167	0.144	12.52	11.46
5	**0.457**	**0.448**	**41.16**	**39.32**	0.168	0.146	11.32	10.32
6	0.444	0.441	39.17	36.23	0.162	0.150	9.96	9.48

AP = Estimated number of clusters calculated by Affinity Propagation

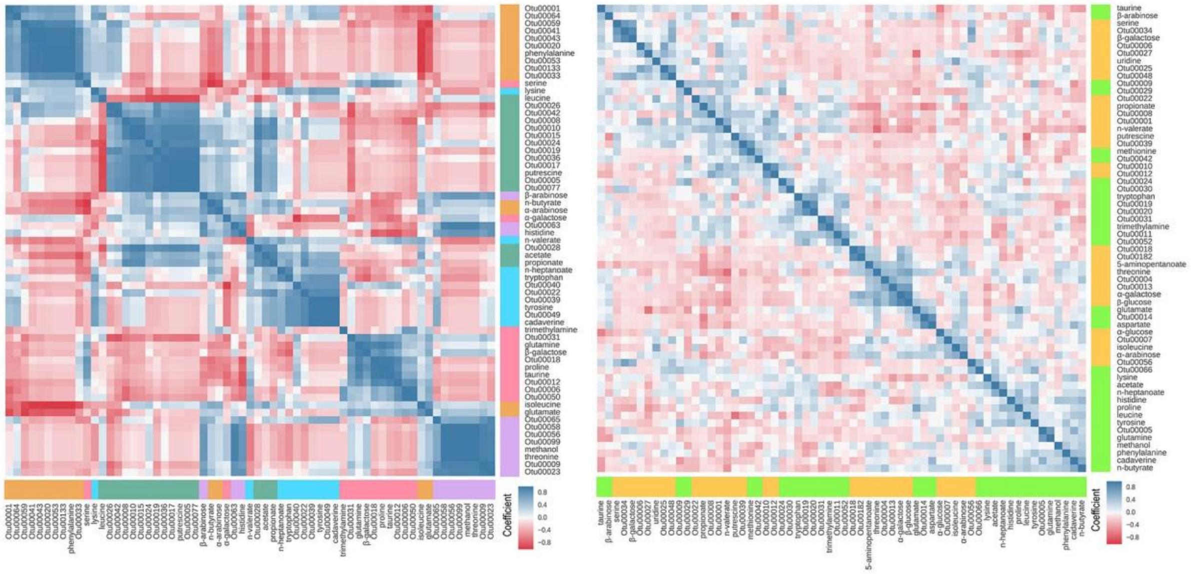

Supplementary figure S6. Correlation heatmaps of fecal bacteria and metabolites. Correlation heatmaps were performed on fecal 16S and NMR merged datasets, both for controls (left) and cirrhotics (right). Pearson coefficient (*r*), ranging from positive (blue) to negative (red) values, was used to build correlation heatmaps (metric=Bray-Curtis, method=complete linkage), while cluster coloring was based on FMCs derived from network analysis (figure 2C, 2D).

SUPPLEMENTARY REFERENCES

1 Schloss PD, Westcott SL, Ryabin T, *et al.* Introducing mothur: Open-Source, Platform-Independent, Community-Supported Software for Describing and Comparing Microbial Communities. *Appl Environ Microbiol* 2009;**75**:7537–41.

2 Quast C, Pruesse E, Yilmaz P, *et al.* The SILVA ribosomal RNA gene database project: improved data processing and web-based tools. *Nucleic Acids Res* 2013;**41**:D590–6.

3 Schloss PD. Evaluating different approaches that test whether microbial communities have the same structure. *ISME J* 2008;**2**:265–75.

4 Segata N, Izard J, Waldron L, *et al.* Metagenomic biomarker discovery and explanation. *Genome Biol* 2011;**12**:R60.

5 Heymann S, Sébastien H. Gephi. In: *Encyclopedia of Social Network Analysis and Mining*. 2014. 612–25.

6 Li M, Wang B, Zhang M, *et al.* Symbiotic gut microbes modulate human metabolic phenotypes. *Proc Natl Acad Sci U S A* 2008;**105**:2117–22.

7 Merico D, Gfeller D, Bader GD. How to visually interpret biological data using networks. *Nat Biotechnol* 2009;**27**:921–4.

8 Berry D, Widder S. Deciphering microbial interactions and detecting keystone species with co-occurrence networks. *Front Microbiol* 2014;**5**:219.

9 Faust K, Sathirapongsasuti JF, Izard J, *et al.* Microbial co-occurrence relationships in the human microbiome. *PLoS Comput Biol* 2012;**8**:e1002606.

10 Lozupone CA, Stombaugh JI, Gordon JI, *et al.* Diversity, stability and resilience of the human gut microbiota. *Nature* 2012;**489**:220–30.

11 Faust K, Raes J. Microbial interactions: from networks to models. *Nat Rev Microbiol* 2012;**10**:538–50.

12 Blondel VD, Guillaume J-L, Lambiotte R, *et al.* Fast unfolding of communities in large networks. *J Stat Mech: Theory Exp* 2008;**2008**:P10008.

13 Lambiotte R, Delvenne J-C, Barahona M. Random Walks, Markov Processes and the Multiscale Modular Organization of Complex Networks. *IEEE Trans Netw Sci Eng*;**1**:76–90.

14 Bahmani B, Moseley B, Vattani A, *et al.* Scalable k-means. *Proceedings VLDB Endowment* 2012;**5**:622–33.

15 Zhang T, Ramakrishnan R, Livny M. BIRCH. *ACM SIGMOD Record* 1996;**25**:103–14.

16 Frey BJ, Dueck D. Clustering by passing messages between data points. *Science* 2007;**315**:972–6.

17 Rousseeuw PJ. Silhouettes: A graphical aid to the interpretation and validation of cluster analysis. *J Comput Appl Math* 1987;**20**:53–65.

18 Calinski T, Harabasz J. A Dendrite Method for Cluster Analysis. *Communications in Statistics - Simulation and Computation* 1974;**3**:1–27.

19 Garreta R, Moncecchi G. *Learning scikit-learn: Machine Learning in Python*. Packt Publishing Ltd 2013.

20 Varoquaux G, Buitinck L, Louppe G, *et al.* Scikit-learn. *GetMobile: Mobile Computing and Communications* 2015;**19**:29–33.

21 Ramsey F, Schafer D. *The Statistical Sleuth: A Course in Methods of Data Analysis*. Cengage Learning 2012.

22 Langille MGI, Zaneveld J, Caporaso JG, *et al.* Predictive functional profiling of microbial communities using 16S rRNA marker gene sequences. *Nat Biotechnol* 2013;**31**:814–21.

23 Parks DH, Tyson GW, Hugenholtz P, *et al.* STAMP: statistical analysis of taxonomic and functional profiles. *Bioinformatics* 2014;**30**:3123–4.

24 Mannina L, Luisa M, Michela C, *et al.* High-Field Nuclear Magnetic Resonance (NMR) Study of Truffles (Tuber aestivum vittadini). *J Agric Food Chem* 2004;**52**:7988–96.

25 Dieterle F, Ross A, Schlotterbeck G, *et al.* Probabilistic quotient normalization as robust method to account for dilution of complex biological mixtures. Application in 1H NMR metabonomics. *Anal Chem* 2006;**78**:4281–90.

26 Hochrein J, Zacharias HU, Taruttis F, *et al.* Data Normalization of (1)H NMR Metabolite Fingerprinting Data Sets in the Presence of Unbalanced Metabolite Regulation. *J Proteome Res* 2015;**14**:3217–28.
